# A living critical interpretive synthesis to yield a framework on the production and dissemination of living evidence syntheses for decision-making

**DOI:** 10.1186/s13012-024-01396-2

**Published:** 2024-09-27

**Authors:** Cristián Mansilla, Qi Wang, Thomas Piggott, Peter Bragge, Kerry Waddell, Gordon Guyatt, Arthur Sweetman, John N. Lavis

**Affiliations:** 1 McMaster Health Forum, McMaster University, 1280 Main St W MML-417, Hamilton, ON L8S 4L6 Canada; 2 Health Policy PhD Program, McMaster University, 1280 Main St W 2C Area, Hamilton, ON L8S 4K1 Canada; 3 Department of Health Research Methods Evidence and Impact, McMaster University, 1280 Main St W 2C Area, Hamilton, ON L8S 4K1 Canada; 4 Peterborough Public Health, 185 King Street, Peterborough, ON K9J 2R8 Canada; 5 Department of Family Medicine, Queens University, 220 Bagot St, Kingston, ON K7L 3G2 Canada; 6 Monash Sustainable Development Institute Evidence Review Service, BehaviourWorks Australia, Monash University, Wellington Rd, Clayton VIC 3800, Melbourne, Australia; 7 Department of Economics, McMaster University, 1280 Main St W Kenneth Taylor Hall Rm. 129, Hamilton, ON L8S 4M4 Canada

**Keywords:** Living evidence syntheses, Living systematic reviews, Evidence-informed health policymaking, Decision-making (4/10)

## Abstract

**Background:**

The COVID-19 pandemic has had an unprecedented impact in the global research production and has also increased research waste. Living evidence syntheses (LESs) seek to regularly update a body of evidence addressing a specific question. During the COVID-19 pandemic, the production and dissemination of LESs emerged as a cornerstone of the evidence infrastructure. This critical interpretive synthesis answers the questions: What constitutes an LES to support decision-making?; when should one be produced, updated, and discontinued?; and how should one be disseminated?

**Methods:**

Searches included the Cochrane Library, EMBASE (Ovid), Health Systems Evidence, MEDLINE (Ovid), PubMed, and Web of Science up to 23 April 2024 and included articles that provide any insights on addressing the compass questions on LESs. Articles were selected and appraised, and their insights extracted. An interpretive and iterative coding process was used to identify relevant thematic categories and create a conceptual framework.

**Results:**

Among the 16,630 non-duplicate records identified, 208 publications proved eligible. Most were non-empirical articles, followed by actual LESs. Approximately one in three articles were published in response to the COVID-19 pandemic. The conceptual framework addresses six thematic categories: (1) what is an LES; (2) what methodological approaches facilitate LESs production; (3) when to produce an LES; (4) when to update an LES; (5) how to make available the findings of an LES; and (6) when to discontinue LES updates.

**Conclusion:**

LESs can play a critical role in reducing research waste and ensuring alignment with advisory and decision-making processes. This critical interpretive synthesis provides relevant insights on how to better organize the global evidence architecture to support their production.

**Trial registration:**

PROSPERO registration: CRD42021241875.

**Supplementary Information:**

The online version contains supplementary material available at 10.1186/s13012-024-01396-2.

Contributions to the literature

- The COVID-19 pandemic positioned living evidence syntheses (LESs) as a key feature of the global evidence architecture.
- This synthesis creates a framework for producing and disseminating LESs for decision-making.
- Six thematic categories were identified: (1) what is an LES; (2) what methodological approaches facilitate LESs production; (3) when to produce an LES; (4) when to update an LES; (5) how to make available the findings of an LES; and (6) when to discontinue updates to an LES.
- This unique conceptual framework can help connect LESs with their role in decision-making processes during health emergencies and in more routine circumstances.

## Background

The COVID-19 pandemic has had an unprecedented impact on the global population. The World Health Organization (WHO) shows that millions of people died since the start of the pandemic, which is confirmed by recent estimates of excess mortality reported by several countries [1]. The COVID-19 pandemic is now seen as the global health event with the greatest consequences to the world’s health in the last century.

The COVID-19 pandemic not only stressed public-health systems; it also stressed the existing research infrastructure. Before the pandemic, researchers had shown a significant increase in research outputs, which had escalated to unprecedented levels, with large variability in value and coordination among evidence producers [2]. Research output accelerated further during the COVID-19 pandemic [3], creating even bigger challenges with research waste on the one hand and significant gaps from the perspective of decision-makers on the other hand.

In this context, decision-makers have faced difficulties in finding and using the best available research evidence to address the specific challenges they face. Leaving aside the complexity of the issues that the COVID-19 pandemic brought to the fore, decision-makers faced additional complexity in understanding and interpreting the evidence that the COVID-19 pandemic elicited [4].

Living evidence syntheses (LESs) are an approach to regularly updating a body of evidence addressing a specific question. LESs were first described in the literature in 2017 [5], and began being produced by Cochrane [6] and other evidence producers before the COVID-19 pandemic started. Complementary to LESs, living guidelines have also been developed and piloted as a valuable approach to provide recommendations. During the pandemic, LESs that produced regularly updated summaries of what was known played an important role in informing decisions. Thus, the production, dissemination, and use of LESs are now considered a key cornerstone of the global evidence architecture [7]. Given the recency of the prominence of LESs, each of these dimensions requires greater conceptual clarity.

We began this synthesis by using a compass question worded as follow: “What, when and why to produce and disseminate living evidence syntheses for decision-making?” (registered in the PROSPERO record). A compass question can, however, be iteratively adjusted as greater conceptual clarity is gained [8]. The final version of the compass question is as follows: “What constitutes an LES to support decision-making? When should one be produced, updated and discontinued, with what methodological support produced and updated, and how should one be disseminated?”.

## Methods

The protocol of this critical interpretive synthesis has been published in PROSPERO (https://www.crd.york.ac.uk/prospero/display_record.php?ID=CRD42021241875) and key details are summarized below. Critical interpretive syntheses are a type of evidence synthesis in which, by doing a critical and interpretive qualitative analysis from the literature, its main objective is to create a conceptual framework to understand a phenomenon of interest [8].

### Search methods

To identify potentially relevant documents, the following bibliographic databases were searched:

- Cochrane Library, including CENTRAL (inception to 23 April 2024)
- Health Systems Evidence (inception to 23 April 2024)
- MEDLINE and EMBASE using Ovid (inception to 23 April 2024)
- PubMed (inception to 23 April 2024)
- Web of Science (inception to 23 April 2024).

The electronic database search was supplemented by examining the references of included articles, and evidence syntheses that were captured in the screening process. Additional file 1 describes the search strategies that were used in each database.

### Study selection

By identifying or examining relationships among relevant considerations, eligible articles provided insights on the production or dissemination of LESs for decision-making. (i.e., eligible articles did not have to be LESs). No restrictions on study design, language, publication type or publication date were applied.

Articles were excluded if they:

- were not LESs, and did not provide insights on LESs;
- were LESs but provide no insights on the production or dissemination of them;
- provide insights but are restricted to evidence-to-decision aspects of living guidelines (whereas we would include papers providing insights applicable to both living guidelines and LESs).

Duplicates were removed using EndNote® and Covidence®. Two members of the research team independently screened all titles and, abstracts. Then, two independent reviewers screened all full texts, resolving disagreements by a third reviewer; reviewers used Covidence® to conduct this process.

### Data extraction

One reviewer extracted the following characteristics from the included articles:

- lead author, month, and year of publication, and citation;
- type of article (LES as declared by the authors; non-LES; empirical article, excluding evidence syntheses; non-empirical article (e.g., commentary or editorial));
- study design and geographical scope for an empirical study, as reported by the authors;
- sector where the article is relevant (following the taxonomy used by the COVID-END (COVID-END was a time-limited network of groups supporting some type of evidence production and uses to work together on how to better inform COVID-19 decision-making) inventory of evidence syntheses [9]: clinical management; public-health measures; health-system arrangements; economic and social responses)
- insights addressing the compass question and its components;
- whether or not the article was produced in the context of the COVID-19 pandemic.

Studies with more than one publication were managed as follows:

- if a published and a pre-print version was available, the peer-reviewed version of the article was considered for extraction;
- if a full paper was linked to a conference abstract that was captured by a search strategy, the full paper was considered for extraction;
- if a published protocol of an evidence synthesis was available, both the protocol and the published version were considered for extraction;
- if updates of an LESs were available, the latest update was considered for extraction and, if any additional insights were found in older updates, they too were considered for extraction.

A data extraction template was first piloted by two authors, and the full data-extraction process was conducted in Microsoft Excel®.

### Quality assessment

Empirical primary studies and evidence syntheses were appraised for their methodological limitations. For primary studies, the mixed methods appraisal tool (MMAT) [10] was used, as it allowed appraisal of a broad range of empirical studies. A single reviewer conducted this appraisal.

Evidence syntheses were evaluated using the AMSTAR instrument [11]. Two reviewers independently conducted this appraisal, discussing any potential conflicts to reach a consensus. When available, the AMSTAR score posted in the COVID-END Inventory, Health Systems Evidence or Social Systems Evidence was used.

Protocols of studies actively underway were not appraised for their methodological limitations.

### Data synthesis

Based on the information collected in the data extraction form, each article was classified according to its contributions to addressing the compass question, and whether or not it provided insights about the production and/or dissemination of living evidence syntheses for decision-making.

Based on all the articles considered as eligible, a conceptual framework was created by conducting a narrative synthesis using a coding strategy from the insights coming from the included documents. This coding was conducted in an interpretive and iterative way, starting by the articles classified as highly relevant in the data-extraction stage. Later, insights from articles in each of the draft thematic categories were incorporated in the framework.

To complement the above, a qualitative analysis was conducted based on discussions that were originated in a listserv that is supported by COVID-END, (two listserv discussions with comments from 15 March until 31 March 2021 with approximately 15 contributions), about the role of living reviews in decision-making. These discussions addressed approaches on how to understand LESs, followed by a question on when updates to an LES should be discontinued.

The insights collected from the literature and the listserv discussion are visually presented in a conceptual framework and are detailed in a set of tables describing the insights collected from the thematic categories that emerged from these data sources.

### Living evidence synthesis strategy

This is a living critical interpretive synthesis. The existing criteria for when a living evidence synthesis is needed [5] were met for this critical interpretive synthesis. First, the issue of living evidence syntheses is clearly an ongoing priority for decision-making. Secondly, while the framework included here is comprehensive, there might be new literature that could lead to adjustments to specific thematic categories, such as new methodological ways to support the production of living evidence syntheses. Finally, at the time of this review, several other living evidence syntheses are ongoing, which may lead to changes in the findings from this critical interpretive synthesis.

The search strategies will be continuously updated every 12 months to check for any potential new articles, and this synthesis will be updated at least three times after its first publication. Insights gained to that point will inform the timing of subsequent updates.

## Results

### Search results

**Fig. 1:**
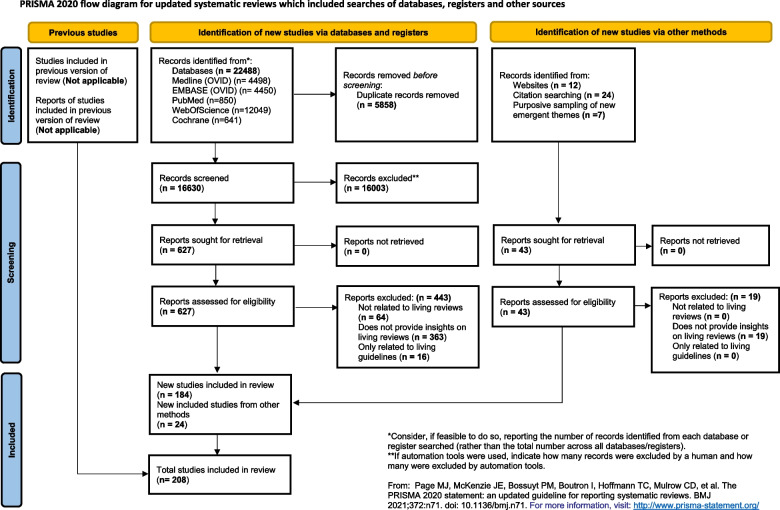
PRISMA diagram showing the review process for selecting the included studies

Among the 22,488 records found, 16,630 non-duplicated abstracts were screened, and 627 full texts were reviewed for a final set of 184 studies described in 281 publications (many of which were updates of LESs). To fill gaps in the conceptual framework, an additional 24 articles were added using a purposive sampling approach from references of the existing articles, which resulted in 208 included articles. See Fig. 1 for the PRISMA flow diagram of the synthesis. Additional file 2 provides a list of the studies excluded in the final stage, along with the reasons for exclusions.

Sixteen conference abstracts proved relevant, but full-text versions of the papers proved unavailable [12–27]; they were included and extracted in their abstract form.

### Description of studies

Table 1 describes the characteristics of the included studies. The majority of articles that provided insights for this synthesis were non-empirical articles, followed by evidence syntheses (living or not). Only a small number of articles were empirical studies that were not evidence syntheses, and 59 (28%) of all studies included were produced in response to the COVID-19 pandemic.

**Table 1:** Description of the included studies

	**N**	**%**
**Type of article**		
Living evidence synthesis	59	28
Non-living evidence synthesis	28	13
Empirical article (not evidence synthesis)	14	7
Non-empirical article (e.g., commentary, editorial, etc.)	109	52
**Produced in response to the COVID-19 pandemic**	59	28
**Sector that is most relevant**^**a**^		
Public-health measures	23	11
Clinical management	84	40
Health-system arrangements	5	2
Economic and social responses	4	2
No particular focus	107	51
**Thematic categories**^**a**^		
1. Definition of an LES	94	45
2. Methods to assess the need and produce an LES	108	51
3. When to produce an LES	70	33
4. When to update an LES	73	35
5. Dissemination of LES findings	70	33
6. When to discontinue updates to an LES	22	11

^a^One article could address more than one thematic category or sector. Percentages could sum more than 100%

In terms of thematic focus, most of the articles did not have a particular focus, followed by articles addressing clinical management issues, and then those focused on public-health measures. Nine articles addressed health-system arrangements and economic and social responses. Finally, thematic categories were relatively equally served in terms of the number of articles, with the exception being when to discontinue updates to an existing LES, an issue addressed by only 22 articles (11%).

Among the small number of empirical studies, the study designs varied (mixed-method, qualitative, and quantitative); one study was conducted in each of Australia [28], Italy [29], and the United States [30], and the fourth was conducted in both Australia and Canada [31], while the remainder did not have a specific geographical scope. Of the 208 articles, 121 were classified as highly relevant mainly based on the importance of their contributions to creating the conceptual framework.

The quality of the evidence syntheses was moderate to high; most were moderate quality in the AMSTAR instrument (4 to 7). The limited number of empirical primary studies showed a wide variation in terms of their methodological limitations but they fill most of the criteria from MMAT. Additional file 3 shows the detail of the AMSTAR scores for evidence syntheses, and methodological limitations for empirical primary studies using the MMAT.

### Results of the coding

Six thematic categories were identified from the data sources within which there were 21 different sub-themes. With the exception of the sub-theme ‘labelling living’ in the thematic category 1 that emerged only from the listserv discussion, the remainder of the thematic categories and sub-themes emerged from both the literature and the listserv discussion.

In conducting the critical analysis, two specific topics emerged as potential controversies or gaps in the literature. First, the definition of what should be considered an update (thematic category 1, sub-theme 2. Updates) was addressed by multiple ideas. Secondly, three specific gaps were found in the critical analysis, and they were filled by purposively sampled literature: (1) when an update was needed, which was filled by literature about when a non-living evidence synthesis needs to be updated; (2) when an issue was a priority for decision-making, which was filled by the agenda setting literature; and (3) the applicability of the findings of a living evidence synthesis for different contexts and issues. Complementary, Table 2 explains how each sub-theme relates to each thematic category, as well as the reference of the papers contributing to each thematic category. Additional file 4 provides a detailed description of how each article contributed to each thematic category and sub-theme, while Additional file 5 provides a more thorough description of each one of the thematic categories.

**Table 2:** Description of the thematic categories and sub-themes that emerged from the literature

	**Thematic categories**					
	**1. Definition of an LES**	**2. Methods to assess the need and produce an LES**	**3. When to produce an LES**	**4. When to update an LES**	**5. Dissemination of the findings of an LES**	**6. When to discontinue updates to an LES**
**General sub-themes**	1.1. What is an LESs: Understanding a living synthesis as a summary of all existing research that is up-to-date at any defined point in time 1.2. What is an update: Understanding what constitutes an ‘update’ in the context of an LES 1.3. Labeling ‘living’: What do we understand by the label ‘living’	2.1. Assessment of the need of an LES: Methods to predict whether: - new literature might change the findings - the likelihoods of the existing findings to change - the value that new information would provide in reducing uncertainty - or how the context and issue might change the applicability of the findings 2.2. Team management: Methods to facilitate team management while producing an LES 2.3. Production: Methods to facilitate the production (searching, selecting studies, extracting data, assessing risk-of-bias and synthesizing data) of an LES	3.1. Types of decision: Alternatives for an evidence producer when starting a new evidence synthesis: - starting a new synthesis -updating a synthesis -making A synthesis living) 3.2. Other elements to consider other elements to be considered when making a decision of when an LES needs to be conducted (e.g., workload, context, etc.)	4.1. Processed involved: Parts of an evidence synthesis that could be updated, including search methods, data synthesis and publication 4.2. Frequency: deciding how frequent an LES needs to be updated, with what support researchers count on making this decision, and the need for researchers to commit to reliable schedules for updates	5.1. Platforms: different options that could be used to make available the findings: -website - scientific journal -interactive platforms 5.2. Structured format: different adaptations to the format of an LES that can be used to streamline dissemination processes 5.3. LES users: types of decision-makers and evidence intermediaries that can use the findings of LESs, and the need for evidence producers to tailor the presentation depending on the type of user 5.4. Speeding-up: strategies that can be used to reduce the time from which findings are available and they are used by decision-makers and evidence intermediaries, including pre-prints, small-indexed publications notifying updates	6.1.Other elements to consider: other elements to be considered when making a decision of when an LES can stop being updated, including the engagement of the synthesis team, and the planned obsolescence of an LES
**Sub-themes associated with ‘triggers’**			3.3. Triggers to produce an LES: - Demand-side triggers: relevancy of the topic for decision-making (i.e., priority issue) - Supply-side triggers: likelihood of the existing evidence to be changed, and the probability that new evidence might change the findings	4.3. Triggers to update an LES: - Demand-side triggers: changes in the priority of an issue for decision-making - Supply-side triggers: changes in the likelihood of the existing evidence to be changed, and the probability that new emerging evidence might change the findings		6.2. Triggers to discontinue an LES: - Demand-side triggers: the issue is no longer a priority, or the research question could be re-framed - Supply-side triggers: no new evidence is expected to be available or the findings of the existing evidence are unlikely to change
**Citations**	[5, 17, 18, 20, 21, 23, 29, 32, 32–119]	[5, 12, 13, 19, 22–29, 31, 37, 38, 40, 43, 44, 46, 47, 50–54, 56, 59, 65, 67–71, 77, 80, 88, 92, 93, 95, 96, 98, 99, 103, 108, 110, 120, 120–182]	[5, 16, 21, 30, 33, 37, 38, 42, 44–49, 52–56, 58, 60, 65, 66, 71, 72, 76, 77, 87, 88, 90, 91, 93, 96–99, 101, 104, 110, 113, 114, 116, 117, 123, 131, 133–136, 140, 141, 148–150, 165, 173, 183–196]	[15–18, 28, 30, 32, 38, 39, 42, 45, 47, 49, 50, 54, 56–58, 62, 66, 69, 71, 73, 76, 86, 88, 92, 93, 96–99, 116, 119, 120, 120, 122, 124, 126, 127, 129–131, 134, 136, 142, 144, 148, 150, 156, 157, 160, 173, 176, 184, 190–194, 196–209]	[5, 29, 34, 37, 39–41, 46, 51–54, 56, 61, 62, 65–67, 70, 72, 73, 78, 80, 83, 87, 92, 93, 95, 96, 98, 99, 103, 104, 106, 108–110, 113, 119, 123–125, 133, 135, 141, 147–150, 163, 169, 171, 177, 195, 196, 203, 206–220]	[16, 30, 37, 37, 38, 50, 55, 56, 66, 70, 88, 91–93, 101, 116, 134, 136, 148, 151, 155, 185, 221]

*LES* Living evidence synthesis

The six thematic categories include 21 sub-themes. In the first thematic category, the definition of LES is separated into what constitutes a living synthesis, what constitutes an update, and the meaning of the label “living”. The second thematic category explains the methods that can be used to assess the need for an LES, how to manage a team conducting an LES, and the methods to facilitate the production of an LES. The third and fourth thematic categories include ‘triggers’ to look for when deciding to produce and update an LES, which are structured into demand-side, supply-side, and other type of triggers. The fifth thematic category describes the platforms and format that an LES can use to disseminate its findings. It also describes the potential users to whom the findings of an LES would be disseminated, as well as ways to speed-up the dissemination of LESs. Finally, the sixth thematic category include ‘triggers’ to look for when deciding to discontinue updates of an LES.

### Conceptual framework

**Fig. 2:**
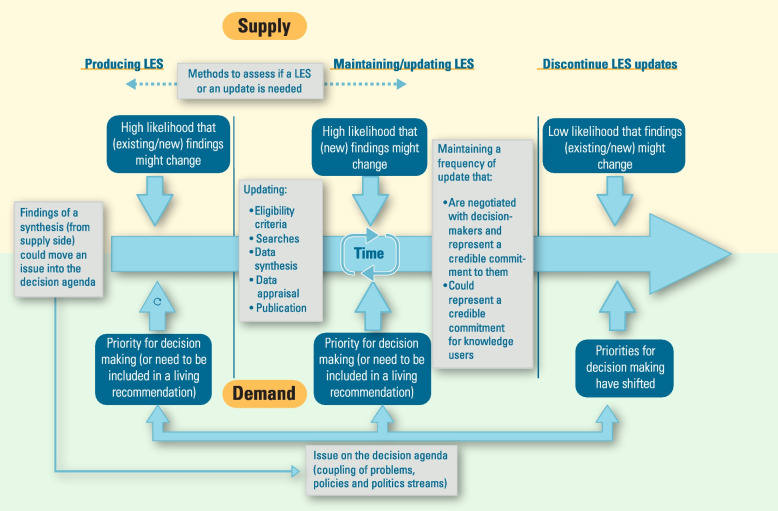
Conceptual framework showing demand and supply triggers in three main stages of living evidence syntheses

Figure 2 shows the conceptual framework created from the thematic categories found in this critical interpretive synthesis. It displays the three main sections of the cycle of an LES (producing, maintaining/updating, and discontinuing updates), which are described in thematic categories 3, 4 and 6. These three sections are arranged around a time axis from left to right, while this axis divides the supply triggers coming from the upper part of the diagram from the demand triggers coming from its lower part.

The demand side is mainly driven by how issues are sitting on the decision agenda, using the Kingdon model of agenda setting, This framework facilitates the understanding of why issues are promoted to the decision agenda in a given time, by coupling the three main streams: the problems stream (i.e., why the problem come to attention), the policies stream (a potential viable solution) and the politics stream (political climate that could be conducive) [30].

This conceptual framework for producing and making available the findings of an LES acknowledges that findings of a given synthesis could also contribute to a rise in attention to an issue, creating a type of feedback. Also, it shows that the conception of an update could come not only from adding new evidence, but also any changes in the underlying structure of an existing synthesis (e.g., eligibility criteria, presentation details, etc.). Additionally, the frequency of updates could be tailored or established in advance, but a negotiation with potential decision-makers and evidence intermediaries is also flagged as important insight. If updating frequently is critical, creating a credible commitment with knowledge users in terms of when to expect new updates is also important. Finally, one important insight gathered from the literature is that the decision of when to start an LES could be similar to the decision regarding when to update one, since every LES will start with a ‘baseline’ synthesis that will be updated regularly. The framework shows a cycle in terms of the need to assess when to update an LES.

## Discussion

### Principal findings

This critical interpretive synthesis considered a broad literature and a series of posts included in a listserv discussion to create a conceptual framework to understand what LESs are, and when and how to produce and disseminate them. The resulting framework (Fig. 2) structured the LES process in three main ‘buckets’: starting an LES, maintaining or updating an LES, and deciding to discontinue updates. It also highlights the main triggers that could inform each stage from the demand and supply sides. While the triggers from the demand side are mainly associated with whether an issue is a priority for decision-making, the triggers from the supply side are associated with the likelihood that the existing body of evidence for a given question might change.

The six thematic categories included 21 sub-themes that were included as part of the analysis reflecting the complexity and the number of different aspects involved in the production and dissemination of LESs. Considering that the first paper on LESs was only published in 2017 [5], this area has grown substantially in complexity in a short period of time. It has also been powered by the COVID-19 pandemic, which established LESs as a key cornerstone of the global evidence architecture [7].

### Findings in relation to other studies

This is the first paper creating a conceptual framework to support the production and dissemination of LESs. While the first paper on LESs was published a number of years ago [5], several efforts to advance thinking and practice of LESs have been undertaken since then, by several evidence producers, including the Cochrane Collaboration [6]. During the COVID-19 pandemic, the number of LESs grew exponentially [3], with most being efforts that could be relied on in terms of their frequency of regular updates, some of them never making it beyond the publication of a protocol to the publication of their first version. The COVID-19 pandemic highlighted the importance of LESs for decision-making as contexts and issues constantly evolved, as did evidence production.

### Strengths and limitations

This paper has four important strengths. First, although the main body of literature came from the health sector, it provides a conceptual framework that is relevant to a variety of decision-makers in different sectors. Secondly, it is designed to be a living CIS that will be updated as soon as new literature provides new insights, keeping the conceptual framework up-to-date. This is particularly relevant as it is expected that as the LESs addressing COVID-19 are discontinued, they may surface new insights from authors to inform the framework produced in this synthesis. Thirdly, the data sources included were exhaustive, using a comprehensive search of the literature combined with an analysis of dedicated insights on the role of LESs for decision-making. Finally, the paper incorporates other conceptual frameworks where relevant (e.g., agenda setting processes), providing a more comprehensive understanding of the complex processes addressed.

This article has limitations. First, this paper focused on the production and dissemination of the findings of an LES. Although the potential uses of LESs for decision-making were partially addressed by considering the demand-side triggers to gather emerging insights from the literature, these were beyond the scope of this paper. Secondly, we mainly found literature that was not empirical. No rigorous evaluations were available that could address the impact of LESs on decision-making. Finally, some parts of the evidence synthesis process were conducted by using only a single reviewer (i.e., data extraction and assessing the methodological limitations of the included articles).

### Implications for policy and practice

This framework can inform decision-makers, evidence intermediaries and evidence producers regarding the role that LESs can play in decision-making processes. On the one hand, LESs can inform decision-makers as well as considerations related to commissioning and setting expectations for LES teams. On the other hand, it can help evidence intermediaries and producers with demand-side considerations related to conducting, updating, or discontinuing updates to an LES, as well as what approaches they can use to facilitate this work.

Evidence producers can use this framework to inform their efforts regarding when to produce an LES. This could help to reduce research waste by facilitating coordination among evidence producers to encourage the production of a suite of high-quality living evidence syntheses on priority topics, as opposed to multiple (sometimes duplicate) initiatives conducting non-living evidence syntheses. However, incentives from funders and academic publications might act as a barrier to reach this goal.

When conducting a living evidence synthesis, evidence producers should transparently report and adhere to their plans regarding update frequencies and how they are planning to be updated. This will help to focus their research funding efforts on topics that would produce sound and relevant LESs.

Additionally, this framework can also be used to consider whether living datasets could be served by this analysis. Hence, the role of living evidence might not necessarily be at the level of syntheses or documents, but also extend to other forms of evidence.

### Implications for future research

Future research efforts should address how LESs could be better structured and organized by evidence producers, intermediaries, and decision-makers to better coordinate their actions to facilitate the effective uses of different types of evidence in the decision-making process. Empirical studies that ask decision-makers, evidence intermediaries and evidence producers about how to advance the usefulness of this framework could provide additional insights by conducting prioritizing exercises (e.g., Delphi studies) or ones that provide qualitative insights (e.g., case study) to test to support evidence producers and intermediaries on when to produce, update and discontinue LESs.

## Conclusion

This critical interpretive synthesis provides a conceptual framework to better display the different elements on how to understand what LESs are, and when and how to produce and disseminate them. Six thematic categories emerged from the literature, highlighting definitions and methods to produce an LES, triggers from the demand and supply side to initiate production, update and discontinue updating LESs, and insights into how to make available findings of an LES. This framework can inform decision-makers, evidence intermediaries and evidence producers to clarify the role that LESs can play in decision-making processes. Future research could advance the usefulness of this framework by testing it and putting it into practice to facilitate the use of LESs in decision-making processes.

## Supplementary Information

Additional file 1. Search strategies: detailed search strategies used in this evidence synthesis.

Additional file 2. Excluded articles. List of articles excluded specifying their reasons.

Additional file 3. Quality of the evidence syntheses included, and methodological limitations of the empirical primary studies included. Appraisal of the quality of the evidence syntheses (using the AMSTAR score), and methodological limitations of primary studies (using MMAT).

Additional file 4. Contribution of each article to the thematic categories. Specification of how each article contributes to each thematic categories and sub-themes identified by the evidence synthesis.

Additional file 5. Detailed description of each thematic category. Detailing and explaining how each thematic category and sub-theme are framed.

## Data Availability

All data generated or analysed during this study are included in this published article [and its supplementary information files].

## Abbreviations

CIS: Critical interpretive synthesis
LES: Living evidence synthesis
