# Supplementary material for: A living critical interpretive synthesis to yield a framework on the production and dissemination of living evidence syntheses for decision-making
Source: Implement Sci. 2024 Sep 27;19:67. doi: 10.1186/s13012-024-01396-2 (PMC11429155; doi:10.1186/s13012-024-01396-2)
Supplement: Supplementary file 1 — Additional file 1. Search strategies: detailed search strategies used in this evidence synthesis. [file 13012_2024_1396_MOESM1_ESM.docx]

# Additional File 1. Search strategies

| Database | Search strategy |
| --- | --- |
| OVID (Medline and EMBASE) | (living or live).mp. adj 4 (reviews or review or evidence or synthes* or meta-analy* or metanaly* or "meta analysis" or "meta analyses" or map or maps or overview* or SR).mp |
| Cochrane | (living or live) NEAR/4 (reviews or review or evidence or synthes* or meta-analy* or metanaly* or "meta analysis" or "meta analyses" or map or maps or overview* or SR) |
| WebofScience (excludes Medline) | TS = ((living OR live) NEAR/4 (reviews OR review OR evidence OR synthes* OR meta-analy* OR metanaly* OR "meta analysis" OR "meta analyses" OR map OR maps OR overview* OR SR))) |
| PubMed | (living or live) n4 reviews or review or evidence or synthes* or meta-analy* or metanaly* or "meta analysis" or "meta analyses" or map or maps or overview* or SR) |
| HealthSystemsEvidence | (living OR live) AND (reviews OR review OR evidence OR synthesis OR syntheses OR meta-analysis OR metanalysis OR "meta analysis" OR "meta analyses" OR map OR maps OR overview OR SR) |
