# Supplementary material for: A living critical interpretive synthesis to yield a framework on the production and dissemination of living evidence syntheses for decision-making
Source: Implement Sci. 2024 Sep 27;19:67. doi: 10.1186/s13012-024-01396-2 (PMC11429155; doi:10.1186/s13012-024-01396-2)
Supplement: Supplementary file 2 — Additional file 2. Excluded articles. List of articles excluded specifying their reasons. [file 13012_2024_1396_MOESM2_ESM.docx]

**LIST OF EXCLUDED ARTICLES**

# Articles excluded because they are not related to living reviews

1. Adusumilli 2022

Afach S, Chaimani A, Evrenoglou T, Penso L, Brouste E, Sbidian E, et al. Meta-analysis results do not reflect the real safety of biologics in psoriasis. The British journal of dermatology. 2021;184:415–24.

1. Al-Inany 2016

Al-Inany HG, Youssef MA, Ayeleke RO, Brown J, Lam WS, Broekmans FJ. Gonadotrophin-releasing hormone antagonists for assisted reproductive technology. The Cochrane database of systematic reviews. 2016;4:CD001750.

1. Anonymous 2008

Anonymous. 2008; Available from: <http://ovidsp.ovid.com/ovidweb.cgi?T=JS&PAGE=reference&D=medp&NEWS=N&AN=21433403>

1. Antonio 2020

Antonio E, Alobo M, Tufet Bayona M, Marsh K, Norton A. Funding and COVID-19 research priorities - are the research needs for Africa being met? AAS open research. 2020;3:56.

1. Badgett 2015

Badgett RG, Vindhyal M, Stirnaman JT, Gibson CM, Halaby R. A Living Systematic Review of Nebulized Hypertonic Saline for Acute Bronchiolitis in Infants. JAMA pediatrics. 2015;169:788–9.

1. Bogdanovic 2022

Bogdanovic T, Miller MC, Blecha L. Electromagnetic counterparts to massive black-hole mergers. Living reviews in relativity. 2022;25:3.

1. Boomsma 2022

Boomsma C, Kamath M, Keay S, Macklon N. Peri‐implantation glucocorticoid administration for assisted reproductive technology cycles. Cochrane Database of Systematic Reviews [Internet]. 2022.

1. Bover 2020

Bover J, Mateu S, DaSilva I, Gracia S, Sanchez-Baya M, Arana C, et al. Evidence in chronic kidney disease-mineral and bone disorder guidelines: Is it time to treat or time to wait? Clinical kidney journal. 2020;13:513–21.

1. Buttigieg 2014

Buttigieg PL, Ramette A. A guide to statistical analysis in microbial ecology: a community-focused, living review of multivariate data analyses. FEMS microbiology ecology. 2014;90:543–50.

1. Cahill 2015

Cahill K, Hartmann-Boyce J, Perera R. Incentives for smoking cessation. Cochrane Database of Systematic Reviews. 2015;

1. Caliesch 2019

Caliesch R, Hilfiker R. Diagnostic test accuracy of clinical tests and ultrasound for the detection of cam and pincer morphology a systematic review. Swiss Medical Weekly. 2019;149:21S.

1. Ciliska 2001

Ciliska D, Mastrilli P, Ploeg J, Hayward S, Brunton G, Underwood J. The effectiveness of home visiting as a delivery strategy for public health nursing interventions to clients in the prenatal and postnatal period: a systematic review. Primary Health Care Research and Development. 2001;2:41–54.

1. Collins 2020

Collins GS, Ma J, Dhiman P. There are no shortcuts in the development and validation of a covid-19 prediction model. Transboundary and emerging diseases [Internet]. 2020; Available from: <http://ovidsp.ovid.com/ovidweb.cgi?T=JS&PAGE=reference&D=emexa&NEWS=N&AN=632855172>

1. Dahal 2021

Dahal P., Singh-Phulgenda S., Ngu R., Rashan S., Brack M., Maguire B., et al. Methodological variations in design and conduct of visceral leishmaniasis clinical trials: A systematic review. Tropical Medicine and International Health. 2021;26:57.

1. Darmon 2017

Darmon SK, Kushnir VA, Barad DH, Albertini DF, Gleicher N. Systematic review of worldwide trends in assisted reproductive technology 2004-2013. Reproductive Biology and Endocrinology. 2017;15:6.

1. Deitz 2021

Deitz C. Living Documents: A Textual Analysis Review. ROMANIAN JOURNAL OF COMMUNICATION AND PUBLIC RELATIONS. 2021;23:81–4.

1. Duarte 2024

Duarte F, Silva S, Oliveira E, da Silva B, de Melo E, Cabral M, et al. Health educational strategies for people living with HIV: scoping review. ACTA PAULISTA DE ENFERMAGEM. 2024;37.

1. East 2019

East CE, Biro MA, Fredericks S, Lau R. Support during pregnancy for women at increased risk of low birthweight babies. Cochrane Database of Systematic Reviews. 2019;

1. Egilstrod 2019

Egilstrod B, Ravn MB, Petersen KS. Living with a partner with dementia: a systematic review and thematic synthesis of spouses’ lived experiences of changes in their everyday lives (vol 23, pg 541, 2018). Aging & mental health. 2019;23:I–I.

1. Enck 2018

Enck P. Living systematic reviews, not only for clinical (placebo) research. Journal of clinical epidemiology. 2018;98:152–3.

1. Farquhar 2012

Farquhar C, Brown J, Marjoribanks J. Laparoscopic drilling by diathermy or laser for ovulation induction in anovulatory polycystic ovary syndrome. The Cochrane database of systematic reviews. 2012;CD001122.

1. Ferrarello 2016

Ferrarello S. Living Evidence. Husserl’s Ethics and Practical Intentionality. 2016;79–100.

1. Ferri 2001

Ferri RS. As I see it: living the evidence. The Journal of the Association of Nurses in AIDS Care : JANAC. 2001;12 Suppl:88–9.

1. Franik 2018

Franik S, Eltrop SM, Kiesel L, Kremer JAM, Farquhar C. Aromatase inhibitors (letrozole) for subfertile women with polycystic ovary syndrome. Cochrane Database of Systematic Reviews. 2018;2018:CD010287.

1. Gazes 2013

Gazes MI, Zeichner J. Onychomycosis in close quarter living review of the literature. Mycoses. 2013;56:610–3.

1. Glasziou 2013

Glasziou P. From mummified guidelines to living evidence-based medicine: three essential tools. Malaysian family physician : the official journal of the Academy of Family Physicians of Malaysia. 2013;8:13–4.

1. Goldsworthy 2018

Goldsworthy S, McGrail S, Regan C, Pawley J, McCormack S, Varughese M. Lean method, live update-real-time librarian support for evidence based radiotherapy protocols. Radiotherapy and Oncology. 2018;127:S618–9.

1. Greca 2023

Greca R, Miranda B, Bertolino A. State of Practical Applicability of Regression Testing Research: A Live Systematic Literature Review. ACM COMPUTING SURVEYS. 2023;55.

1. Hanratty 2023

Hanratty J, Miller S, Rodriguez L, Connolly P, Roberts J, Sloan S, et al. UPDATED PROTOCOL: Universal school-based programmes for improving social and emotional outcomes in children aged 3-11 years: An evidence and gap map. CAMPBELL SYSTEMATIC REVIEWS. 2023;19.

1. Harrington 2017

Harrington L. Closing the Science-Health Gap With Technology: Evidence-Based Living. AACN advanced critical care. 2017;28:102–6.

1. Hashimoto 2021

Hashimoto S., Yoshizaki K., Uno K., Kitajima H., Arai T., Tamura Y., et al. Prompt Reduction in CRP, IL-6, IFN-gamma, IP-10, and MCP-1 and a Relatively Low Basal Ratio of Ferritin/CRP Is Possibly Associated With the Efficacy of Tocilizumab Monotherapy in Severely to Critically Ill Patients With COVID-19. Frontiers in Medicine. 2021;8:734838.

Hashimoto S, Yoshizaki K, Uno K, Kitajima H, Arai T, Tamura Y, et al. Data_Sheet_1_Prompt Reduction in CRP, IL-6, IFN-gamma, IP-10, and MCP-1 and a Relatively Low Basal Ratio of Ferritin/CRP Is Possibly Associated With the Efficacy of Tocilizumab Monotherapy in Severely to Critically Ill Patients With COVID-19.docx. Figshare. 2021.

1. JacobsonVann 2005

Jacobson Vann JC, Szilagyi P. Patient reminder and recall systems to improve immunization rates. Cochrane Database of Systematic Reviews. 2005;

1. Janiaud 2020

Janiaud P., Hemkens L.G., Ioannidis J.P.A. Challenges and Lessons Learned From COVID-19 Trials: Should We Be Doing Clinical Trials Differently? Canadian Journal of Cardiology. 2021;37:1353–64.

1. Janni 2022

Janni W., Muller V., Schilling J., Lindenmaier P., Jaeger A. Recommendation of the AGO Breast Committee for HER2-Positive Breast Cancer - Real World Data on Implementation and Adherence. Oncology Research and Treatment. 2022;45:18.

1. Jayaram 2019

Jayaram H, Srikantha N, Clarke J. Establishing a New Gold Standard: improving Outcomes in Trabeculectomy Surgery. Investigative ophthalmology & visual science [Internet]. 2019;60. Available from: <https://www.cochranelibrary.com/central/doi/10.1002/central/CN-02007036/full>

1. Jefferson 2018

Jefferson T, Rivetti A, Di Pietrantonj C, Demicheli V. Vaccines for preventing influenza in healthy children. Cochrane Database of Systematic Reviews. 2018;2018:CD004879.

1. Karakulah 2012

Karakulah G, Suner A, Adlassnig K-P, Samwald M. A data-driven living review for pharmacogenomic decision support in cancer treatment. Studies in health technology and informatics. 2012;180:688–92.

1. Kendel 1995

Kendel DA. Living Evidence. Humane Medicine. 1995;11:133–133.

1. Li 2022

Li X., Zhang A., Al-Zaidy R., Rao A., Baral S., Bao L., et al. Automating document classification with distant supervision to increase the efficiency of systematic reviews: A case study on identifying studies with HIV impacts on female sex workers. PLoS ONE. 2022;17:e0270034.

1. Lynch 2021

Lynch I., Nymark P., Doganis P., Gulumian M., Yoon T.-H., Martinez D.S.T., et al. Methods, models, mechanisms and metadata: Introducing the Nanotoxicology collection at F1000Research. F1000Research. 2021;10:1196.

1. Makke 2024

Makke N, Chawla S. Interpretable scientific discovery with symbolic regression: a review. ARTIFICIAL INTELLIGENCE REVIEW. 2024;57.

1. Matl 2017

Matl S, Brosig R, Baust M, Demirci S, Navab N. Vascular image registration techniques: A living review. Medical image analysis. 2017;35:1–17.

1. McNaughton 2010

McNaughton V, Canadian Association for Enterostomal Therapy ECFBPRP, Brown J, Hoeflok J, Martins L, McNaughton V, et al. Summary of best practice recommendations for management of enterocutaneous fistulae from the Canadian Association for Enterostomal Therapy ECF Best Practice Recommendations Panel. Journal of wound, ostomy, and continence nursing : official publication of The Wound, Ostomy and Continence Nurses Society. 2010;37:173–84.

1. Medley 2018

Medley N, Vogel JP, Care A, Alfirevic Z. Interventions during pregnancy to prevent preterm birth: an overview of Cochrane systematic reviews. Cochrane Database of Systematic Reviews [Internet]. 2018; Available from: <http://dx.doi.org/10.1002/14651858.CD012505.pub2>

1. Meng 2000

Meng ID, Grantz D, Kahlon M, Fields HL. AddictionScience.org: interactive Web-based dynamic reviews. Society for Neuroscience Abstracts. 2000;26:Abstract No.-21.8.

1. Mufarrih 2024

Mufarrih SH, Haider S, Qureshi NQ, Khan MS, Kazimuddin M, Akbar MS, et al. Distal Versus Proximal Radial Arterial Access for Percutaneous Coronary Angiography and Intervention: Updated Meta-Analysis of Randomized Controlled Trials. Am J Cardiol. 2024;218:34–42.

1. Norman 2022

Norman G, Shi C, Goh E, Murphy E, Reid A, Chiverton L, et al. Negative pressure wound therapy for surgical wounds healing by primary closure. Cochrane Database of Systematic Reviews [Internet]. 2022; Available from: <http://dx.doi.org/10.1002/14651858.CD009261.pub7>

1. Nuevo-Ortega 2022

Nuevo-Ortega P., Reina-Artacho C., Dominguez-Moreno F., Becerra-Munoz V.M., Ruiz-Del-Fresno L., Estecha-Foncea M.A. Prognosis of COVID-19 pneumonia can be early predicted combining Age-adjusted Charlson Comorbidity Index, CRB score and baseline oxygen saturation. Scientific reports. 2022;12:2367.

1. Nunn 2022

Nunn GA. THE LIVING RULES OF EVIDENCE. UNIVERSITY OF PENNSYLVANIA LAW REVIEW. 2022;170:937–90.

1. O'Connor 2017

O’Connor AM, Auvermann BW, Dzikamunhenga RS, Glanville JM, Higgins JPT, Kirychuk SP, et al. Updated systematic review: associations between proximity to animal feeding operations and health of individuals in nearby communities. Systematic reviews. 2017;6:86.

1. Palacios-Abrantes 2021

Palacios-Abrantes J, Cisneros-Montemayor AM, Cisneros-Mata MA, Rodriguez L, Arreguin-Sanchez F, Aguilar V, et al. Data from: A metadata approach to evaluate the state of ocean knowledge: strengths, limitations, and application to Mexico. Scholars Portal Dataverse. 2021.

1. Pandey 2021

Pandey AK, Mudgil N, Wadgave Y, Mishra SS. Corneal transplantation during COVID-19 pandemic: need for special considerations-A live review. Aims Public Health. 2021;8:186–95.

1. Pinte 2020

Pinte L, Baicus C. Pancreatic involvement in SARS-CoV-2: case report and living review. Journal of gastrointestinal and liver diseases : JGLD. 2020;29:275–6.

1. Raposo 2024

Raposo N, Periole C, Planton M. In-vivo diagnosis of cerebral amyloid angiopathy: an updated review. CURRENT OPINION IN NEUROLOGY. 2024;37:19–25.

1. Richards 2013

Richards D. Evidence Live 2013. Evidence-based dentistry. 2013;14:34.

1. Soll 2020

Soll RF, Ovelman C, McGuire W. The future of Cochrane Neonatal. Early human development. 2020;150:105191.

1. Sopinka 2020

Sopinka NM, Coristine LE, DeRosa MC, Rochman CM, Owens BL, Cooke SJ. Envisioning the scientific paper of the future. Facets. 2020;5:1–16.

1. Thomas 2010

Thomas RE, Lorenzetti DL. Interventions to increase influenza vaccination rates of those 60 years and older in the community. Cochrane Database of Systematic Reviews [Internet]. 2018; Available from: <http://dx.doi.org/10.1002/14651858.CD005188.pub4>

Thomas RE, Russell M, Lorenzetti D, Thomas RE. Interventions to increase influenza vaccination rates of those 60 years and older in the community. Cochrane Database of Systematic Reviews. 2010;

1. Wahabi 2018

Wahabi HA, Fayed AA, Esmaeil SA, Bahkali KH. Progestogen for treating threatened miscarriage. Cochrane Database of Systematic Reviews [Internet]. 2018; Available from: <http://dx.doi.org/10.1002/14651858.CD005943.pub5>

1. Weiss 2017

Weiss D. Living Evidence. Archaeology. 2017;70:15–15.

1. Wheary 1998

Wheary J, Wild L, Weyher C, Schutz B. Thinking in electronic terms. Socioeconomic Dimensions of Electronic Publishing Workshop - Proceedings: Meeting the Needs of the Engineering and Scientific Communities. 1998;83–7.

1. Whittaker 2019

Whittaker R, McRobbie H, Bullen C, Rodgers A, Gu Y, Dobson R. Mobile phone text messaging and app-based interventions for smoking cessation. Cochrane Database of Systematic Reviews. 2019;

1. Wilson 2021

Wilson E., Cruz F., Ghanawi J., Liao J., Mccann S., Macleod M., et al. Developing a living evidence summary of in vivo ischaemic stroke research. European Stroke Journal. 2021;6:471–2.

1. Zakher 2014

Zakher B, Cantor AG, Pappas M, Daeges M, Nelson HD. Screening for Gonorrhea and Chlamydia: A Systematic Review for the U.S. Preventive Services Task Force. Annals of Internal Medicine. 2014;161:884.

# Articles excluded because of they do not insights on living evidence syntheses

1. Addis 2021

Addis A., Amato L., Cruciani F., Saulle R., De Crescenzo F., Mitrova Z., et al. The Standard of Care Definitions on COVID-19 Pharmacological Clinical Trials: A Systematic Review. Frontiers in Pharmacology. 2021;12:749514.

Addis A, Amato L, Cruciani F, Saulle R, De Crescenzo F, Mitrova Z, et al. DataSheet1_The Standard of Care Definitions on COVID-19 Pharmacological Clinical Trials: A Systematic Review.pdf. Figshare. 2021.

1. Afonso 2023

Afonso J, Olivares-Jabalera J, Fernandes R, Clemente F, Rocha-Rodrigues S, Claudino J, et al. Effectiveness of Conservative Interventions After Acute Hamstrings Injuries in Athletes: A Living Systematic Review. SPORTS MEDICINE. 2023;53:615–35.

1. Agarwal 2022

Agarwal A, Basmaji J, Fernando SM, Ge FZ, Xiao Y, Faisal H, et al. Administration of Parenteral Vitamin C in Patients With Severe Infection: Protocol for a Systematic Review and Meta-analysis. JMIR research protocols. 2022;11:e33989.

1. Ahdesmaki 2021

Ahdesmaki O., Ainsworth C., Bell J., Hawe E. PNS7 Ongoing SLRs When Considering HTA Submissions: Rewards and Avoidable Pitfalls. Value in Health. 2021;24:S174.

1. Ajiji 2021

Ajiji P., Cottin J., Ripoche E., Cucherat M., Maison P. Comparison of a semi-automated meta-analysis versus conventional meta-analysis: A study protocol. Fundamental and Clinical Pharmacology. 2021;35:176.

1. AlAttar 2017

Al Attar L, Nehme F, Rowe K, Badgett R. Gastrointestinal bleed: Approach to minimize blood transfusions. American Journal of Gastroenterology. 2017;112:S621.

1. Alderson 2021

Alderson J, Batchelor V, O’Hanlon M, Cifuentes L, Richter FC, Kopycinski J, et al. Overview of approved and upcoming vaccines for SARS-CoV-2: a living review. Oxford open immunology. 2021;2:iqab010.

1. Allan 2020

Allan S. User-Led Digital Interventions for Psychosis “Living” Systematic Review. Open Science Framework. 2020;

1. Allida 2023

Allida S, Hsieh C-F, Cox K, Patel K, Rouncefield-Swales A, Lightbody C, et al. Pharmacological, non‐invasive brain stimulation and psychological interventions, and their combination, for treating depression after stroke. Cochrane Database of Systematic Reviews [Internet]. 2023; Available from: http://dx.doi.org/10.1002/14651858.CD003437.pub5

1. Allin 2021

Allin E, Nama N, Irvine MA, Pawliuk C, Wright M, Carwana M. Conservative and surgical modalities in the management of paediatric parapneumonic effusion and empyema: a protocol for a living systematic review and network meta-analysis. BMJ open. 2021;11:e045010.

InsightScope Team, Elviro C, Longcroft-Harris B, Allin E, Leache L, Woo K, et al. Conservative and Surgical Modalities in the Management of Pediatric Parapneumonic Effusion and Empyema A Living Systematic Review and Network Meta-Analysis. CHEST. 2023;164:1125–38.

1. Allotey 2022

Allotey J., Chatterjee S., Kew T., Gaetano A., Stallings E., Fernandez-Garcia S., et al. SARS-CoV-2 positivity in offspring and timing of mother-to-child transmission: Living systematic review and meta-analysis. The BMJ. 2022;e067696.

Allotey. Update to living systematic review on SARS-CoV-2 positivity in offspring and timing of mother-to-child transmission. BMJ-BRITISH MEDICAL JOURNAL. 2024;384.

1. Altannit 2021

Altannir M, Altannir Y, Tleyjeh R, Tleyjeh IM, Kashour Z, Damlaj M, et al. Efficacy and safety of tocilizumab in COVID-19 patients: a living systematic review and meta-analysis. Clinical Microbiology and Infection. 2021;27:215–27.

1. AmerCollPhys 2023

Amer Coll Phys, Qaseem A, Yost J, Miller M, Andrews R, Jokela J, et al. Outpatient Treatment of Confirmed COVID-19: Living, Rapid Practice Points From the American College of Physicians (Version 1). ANNALS OF INTERNAL MEDICINE. 2023;176:115-+.

1. AmorimDosSantos 2021

Amorim Dos Santos J., Normando A.G.C., Carvalho da Silva R.L., Acevedo A.C., De Luca Canto G., Sugaya N., et al. Oral Manifestations in Patients with COVID-19: A 6-Month Update. Journal of dental research. 2021;100:1321–9.

Amorim Dos Santos J, Carvalho da Silva RL, Acevedo AC, Guerra ENS, Normando AGC, Santos-Silva AR, et al. Oral Manifestations in Patients with COVID-19: A Living Systematic Review. Journal of dental research. 2021;100:141–54.

1. Anand 2024

Anand A, Vialard F, Esmail A, Ahmad Khan F, O’Byrne P, Routy J-P, et al. Self-tests for COVID-19: What is the evidence? A living systematic review and meta-analysis (2020-2023). PLOS Glob Public Health. 2024;4:e0002336.

1. Andrenelli 2020

Andrenelli E., Negrini F., DE Sire A., Patrini M., Lazzarini S.G., Ceravolo M.G. Rehabilitation and COVID-19: update of the rapid living systematic review by Cochrane Rehabilitation Field as of February 28, 2021. European journal of physical and rehabilitation medicine. 2021;57:481–4.

Andrenelli E., Negrini F., de Sire A., Lazzarini S.G., Patrini M., Ceravolo M.G. Rehabilitation and COVID-19: update of the rapid living systematic review by Cochrane Rehabilitation Field as of October 31st, 2021. European journal of physical and rehabilitation medicine. 2022;58:153–6.

Andrenelli E, Ceravolo MG, Negrini F, De Sire A, Patrini M, Lazzarini SG. Rehabilitation and COVID-19: a rapid living systematic review 2020 by Cochrane Rehabilitation Field. Update as of September 30th, 2020. European journal of physical and rehabilitation medicine. 2020;56:846–52.

Ceravolo M.G., Andrenelli E., Arienti C., Cote P., DE Sire A., Iannicelli V., et al. Rehabilitation and COVID-19: rapid living systematic review by Cochrane Rehabilitation Field - third edition. Update as of June 30th, 2021. European journal of physical and rehabilitation medicine. 2021;57:850–7.

Ceravolo MG, Andrenelli E, de Sire A, Negrini F, Negrini S. Systematic rapid “living” review on rehabilitation needs due to COVID-19: update to March 31st, 2020. European journal of physical and rehabilitation medicine. 2020;56:347–53.

Ceravolo MG, Arienti C, de Sire A, Andrenelli E, Negrini F, Lazzarini SG, et al. Rehabilitation and COVID-19: the Cochrane Rehabilitation 2020 rapid living systematic review. European journal of physical and rehabilitation medicine. 2020;56:642–51.

de Sire A., Andrenelli E., Negrini F., Iannicelli V., Lazzarini S.G., Patrini M., et al. Rehabilitation and COVID-19: update of the rapid living systematic review by Cochrane Rehabilitation Field as of August 31st, 2021. European journal of physical and rehabilitation medicine. 2021;57:1045–8.

de Sire A., Andrenelli E., Negrini F., Lazzarini S.G., Cordani C., Ceravolo M.G. Rehabilitation and COVID-19: update of the rapid living systematic review by Cochrane Rehabilitation Field as of February 28th, 2022. European journal of physical and rehabilitation medicine. 2022;58:498–501.

de Sire A, Andrenelli E, Ceravolo MG, Negrini F, Lazzarini SG, Patrini M. Rehabilitation and COVID-19: the Cochrane Rehabilitation 2020 rapid living systematic review. Update as of August 31st, 2020. European journal of physical and rehabilitation medicine. 2020;56:839–45.

de Sire A, Andrenelli E, Ceravolo MG, Negrini F, Negrini S. Systematic rapid living review on rehabilitation needs due to COVID-19: update as of April 30th, 2020. European journal of physical and rehabilitation medicine. 2020;56:354–60.

de Sire A, Andrenelli E, Ceravolo MG, Negrini F, Patrini M, Lazzarini SG. Rehabilitation and COVID-19: a rapid living systematic review by Cochrane Rehabilitation Field updated as of December 31st, 2020 and synthesis of the scientific literature of 2020. European journal of physical and rehabilitation medicine [Internet]. 2021; Available from: <http://ovidsp.ovid.com/ovidweb.cgi?T=JS&PAGE=reference&D=emexb&NEWS=N&AN=634334257>

Negrini F., de Sire A., Andrenelli E., Lazzarini S.G., Patrini M., Ceravolo M.G. Rehabilitation and COVID-19: update of the rapid living systematic review by Cochrane Rehabilitation Field as of April 30, 2021. European journal of physical and rehabilitation medicine. 2021;57:663–7.

Negrini F., de Sire A., Andrenelli E., Lazzarini S.G., Patrini M., Ceravolo M.G. Rehabilitation and COVID-19: update of the rapid living systematic review by Cochrane Rehabilitation Field as of December 31st, 2021. European journal of physical and rehabilitation medicine. 2022;58:328–31.

Negrini F, de Sire A, Andrenelli E, Lazzarini SG, Patrini M, Ceravolo MG. Rehabilitation and COVID-19: the Cochrane Rehabilitation 2020 rapid living systematic review. Update as of July 31st, 2020. European journal of physical and rehabilitation medicine. 2020;56:652–7.

Negrini F, de Sire A, Andrenelli E, Lazzarini SG, Patrini M, Ceravolo MG. Rehabilitation and COVID-19: a rapid living systematic review 2020 by Cochrane Rehabilitation Field. Update as of October 31st, 2020. European journal of physical and rehabilitation medicine. 2021;57:166–70.

Int Multiprofessional Steering, Arienti C, Lazzarini S, Andrenelli E, Cordani C, Negrini F, et al. Rehabilitation and COVID-19: systematic review by Cochrane Rehabilitation. EUROPEAN JOURNAL OF PHYSICAL AND REHABILITATION MEDICINE. 2023;59:800–18.

1. Ang 2022

Ang L., Song E., Hu X.-Y., Lee H.W., Chen Y., Lee M.S. Herbal Medicine Intervention for the Treatment of COVID-19: A Living Systematic Review and Cumulative Meta-Analysis. Frontiers in Pharmacology. 2022;13:906764.

1. Anonymous 2020a

Anonymous. Erratum: Update to living systematic review (BMJ (Clinical research ed.) (2020) 369 (m1328)). BMJ (Clinical research ed). 2020;369:m2204.

1. Anonymous 2020b

Anonymous. Erratum: Interventions for treatment of COVID-19: A living systematic review with meta-analyses and trial sequential analyses (The LIVING Project) (PLoS Med (2020) 17: 9 (e1003293) 10.1371/journal.pmed.1003293). PLoS medicine. 2020;17:e1003517.

1. Anonymous 2020c

Anonymous. Update to living systematic review. BMJ (Clinical research ed). 2020;369:m2204.

1. Anonymous 2020d

Anonymous. 2020; Available from: <http://ovidsp.ovid.com/ovidweb.cgi?T=JS&PAGE=reference&D=medp&NEWS=N&AN=33764719>

1. Anonymous 2021a

Anonymous. Erratum: Accuracy of novel antigen rapid diagnostics for SARS-CoV-2: A living systematic review and meta-analysis (PLoS Med (2021) 18:8 (e1003735) DOI: 10.1371/journal.pmed.1003735). PLoS Medicine. 2021;18:e1003825.

1. Anonymous 2021b

Anonymous. Erratum: COVID-19 in Health-care workers: A living systematic review and meta-analysis of prevalence, risk factors, clinical characteristics, and outcomes (American Journal of Epidemiology (2021) 190:1 (161-175) DOI: 10.1093/aje/kwaa191). American journal of epidemiology. 2021;190:187.

1. Anonymous 2021c

Anonymous. RE: “COVID-19 IN HEALTH-CARE WORKERS: A LIVING SYSTEMATIC REVIEW AND META-ANALYSIS OF PREVALENCE, RISK FACTORS, CLINICAL CHARACTERISTICS, AND OUTCOMES.” American journal of epidemiology. 2021;190:187.

1. Anselmi 2020

Anselmi GD, Ortiz-Munoz LE, Ferrer MBM, Acuna MP, Rada G. Sexual transmission of SARS-CoV-2 virus and its role in the spread of COVID-19: A living systematic review. Open Science Framework. 2020;

1. Anesms 2021

Ansems K, Grundeis F, Dahms K, Mikolajewska A, Thieme V, Piechotta V, et al. Remdesivir for the treatment of COVID‐19. Cochrane Database of Systematic Reviews [Internet]. 2021; Available from: <http://dx.doi.org/10.1002/14651858.CD014962>

1. ArcePardo 2021

Arce Pardo S., Lai S., Ortiz-Munoz L., Bravo-Jeria R., Verdugo-Paiva F., Rada G. Pulmonary rehabilitation for COVID-19: A living systematic review protocol. Medwave. 2021;21:e8224.

1. Arienti 2021

Arienti C, Kiekens C, Bettinsoli R, Engkasan JP, Frischknecht R, Gimigliano F, et al. Cochrane Rehabilitation: 2020 annual report. European journal of physical and rehabilitation medicine. 2021;57:303–8.

1. Arkhipova-Jenkins 2021

Arkhipova-Jenkins I, Armstrong C, Gean E, Paynter RA, Helfand M, Anderson J, et al. Antibody Response After SARS-CoV-2 Infection and Implications for Immunity : A Rapid Living Review. Annals of internal medicine [Internet]. 2021; Available from: <http://ovidsp.ovid.com/ovidweb.cgi?T=JS&PAGE=reference&D=emexb&NEWS=N&AN=634576840>

1. Armon 2021

Armon S., Campbell F., Cierco R., Cree I., Indave I. Mapping the evidence for the WHO Classification of Tumours: A living evidence gap map by tumour type (WCT-EVI-MAP). Virchows Archiv. 2021;479:S37.

1. Asiimwe 2021

Asiimwe I.G., Pushpakom S., Turner R.M., Kolamunnage-Dona R., Jorgensen A.L., Pirmohamed M. Cardiovascular drugs and COVID-19 clinical outcomes: A living systematic review and meta-analysis. British Journal of Clinical Pharmacology. 2021;87:4534–45.

1. Assimwe 2022

Asiimwe I.G., Pushpakom S.P., Turner R.M., Kolamunnage-Dona R., Jorgensen A.L., Pirmohamed M. Cardiovascular drugs and COVID-19 clinical outcomes: a systematic review and meta-analysis of randomized controlled trials. British Journal of Clinical Pharmacology [Internet]. 2022; Available from: <http://onlinelibrary.wiley.com/journal/10.1111/(ISSN)1365-2125>

1. Au 2022

Au W.Y., Cheung P.P.-H. Effectiveness of heterologous and homologous covid-19 vaccine regimens: Living systematic review with network meta-analysis. The BMJ. 2022;e069989.

Anonymous. Update to living systematic review on effectiveness of heterologous and homologous covid-19 vaccine regimens. BMJ (Clinical research ed). 2022;379:o2865.

1. Ayers 2023

Ayers C, Kansagara D, Lazur B, Fu R, Kwon A, Harrod C. Effectiveness and Safety of Treatments to Prevent Fractures in People With Low Bone Mass or Primary Osteoporosis A Living Systematic Review and Network Meta-analysis for the American College of Physicians. ANNALS OF INTERNAL MEDICINE. 2023;176.

1. Bach-Mortensen 2021

Bach-Mortensen A, Esposti MD, Verboom B, Movsisyan A. OWNERSHIP AND COVID-19 IN CARE HOMES FOR OLDER PEOPLE: A LIVING SYSTEMATIC REVIEW OF OUTBREAKS, INFECTIONS, AND MORTALITY. JOURNAL OF EPIDEMIOLOGY AND COMMUNITY HEALTH. 2021;75:A2–A2.

1. Bagg 2018

Bagg MK, McAuley JH. Correspondence: Living systematic reviews. Journal of physiotherapy. 2018;64:133.

1. Baladia 2020

Baladia E, Pizarro AB, Ortiz-Munoz L, Rada G. Vitamin C for COVID-19: A living systematic review. Vitamin C for COVID-19: A living systematic review. 2020;20:e7978.

1. Barbosa 2023

Barbosa M, Canan A, Xi Y, Litt H, Diercks D, Abbara S, et al. Comparative Effectiveness of Coronary CT Angiography and Standard of Care for Evaluating Acute Chest Pain: A Living Systematic Review and Meta-Analysis. RADIOLOGY-CARDIOTHORACIC IMAGING. 2023;5.

1. Barnes 2023

Barnes C, Turon H, McCrabb S, Hodder R, Yoong S, Stockings E, et al. Interventions to prevent or cease electronic cigarette use in children and adolescents. Cochrane Database of Systematic Reviews [Internet]. 2023; Available from: http://dx.doi.org/10.1002/14651858.CD015511.pub2

1. Batista 2022

Batista D.R., Floriano I., Silvinato A., Bacha H.A., Barbosa A.N., Tanni S.E., et al. Use of anticoagulants in patients with COVID-19: a living systematic review and meta-analysis. Jornal Brasileiro de Pneumologia. 2022;48:e20220041.

Batista D, Floriano I, Silvinato A, Bacha H, Barbosa A, Tanni S, et al. Use of anticoagulants in patients with COVID-19: an update of a living systematic review and meta-analysis. JORNAL BRASILEIRO DE PNEUMOLOGIA. 2023;49.

1. Beresford 2022

Beresford L., Evans C., Bryan G., Fulbright H., Phillips B., Morgan J. SYSTEMATIC REVIEW OF EARLY PHASE TRIALS FOR CHILDREN AND YOUNG PEOPLE WITH RELAPSED AND REFRACTORY RHABDOMYOSARCOMA: THE REFORMS-SR PROJECT. Pediatric Blood and Cancer. 2022;69:S255.

1. Bhimraj 2022

Bhimraj A, Morgan R, Shumaker A, Baden L, Cheng V, Edwards K, et al. Infectious Diseases Society of America Guidelines on the Treatment and Management of Patients With Coronavirus Disease 2019 (COVID-19). CLINICAL INFECTIOUS DISEASES. 2022;

1. Bick 2020

Bick D FJ Chang Y-S, Sutcliffe P, Hillman S, Chien LY. Barriers and facilitators to optimizing parents’, carers’ and healthcare workers’ infant feeding hygiene practices to prevent transmission of respiratory infectious diseases, including COVID-19: A living systematic review. PROSPERO. 2020.

1. Bielski 2023

Bielski K, Pruc M, Rafique Z, Peacock F, Chmielewski J, Panasiuk L, et al. Uncovering the effects of COVID-19 on in-hospital cardiac arrest - a living systematic review and meta-analysis. ANNALS OF AGRICULTURAL AND ENVIRONMENTAL MEDICINE. 2023;30:498–504.

1. BinRiaz 2023

Bin Riaz I, Naqvi S, He H, Siddiqi R, Asghar N, Ravi P, et al. A living interactive systematic review and network meta-analysis evaluating systemic therapies in metastatic castration-sensitive prostate cancer (mCSPC). JOURNAL OF CLINICAL ONCOLOGY. 2023;41.

1. Blix 2024

Blix E, Brurberg K, Reierth E, Reinar L, Oian P. ST waveform analysis versus cardiotocography alone for intrapartum fetal monitoring: An updated systematic review and meta-analysis of randomized trials. ACTA OBSTETRICIA ET GYNECOLOGICA SCANDINAVICA. 2024;103:437–48.

1. Boet 2022

Boet S., Etherington C., Ghanmi N., Ioudovski P., Tricco A.C., Sikora L., et al. Efficacy and safety of hyperbaric oxygen treatment to treat COVID-19 pneumonia: a living systematic review update. Diving and hyperbaric medicine. 2022;52:126–35.

1. Bonardi 2020

Bonardi O, Azar M, He C, Sun Y, Krishnan A, Thombs-Vite I, et al. Curating evidence on mental health during COVID-19: A living systematic review. Journal of psychosomatic research. 2020;133:110113.

1. Bond 2021

Bond M. Schools and emergency remote education during the COVID-19 pandemic dataset. Mendeley Data. 2021;2.

1. Bongomin 2023

Bongomin F, Olum R, Kibone W, Namusobya M, van Rhijn N, Denning D. Prevalence of chronic pulmonary aspergillosis along the continuum of pulmonary tuberculosis care: A protocol for a living systematic review and meta-analysis. PLOS ONE. 2023;18.

1. Bookwalter 2023

Bookwalter C. Caring for High-Risk Ambulatory Adult Patients. US PHARMACIST. 2023;48:51–5.

1. Boucher 2023

Boucher E, Cao C, D’Mello S, Duarte N, Donnici C, Bennett G, et al. Occupation and SARS-CoV-2 seroprevalence studies: a systematic review. BMJ OPEN. 2023;13.

1. Bougioukas 2023

Bougioukas K, Pamporis K, Vounzoulaki E, Karagiannis T, Haidich A. Types and associated methodologies of overviews of reviews in health care: a methodological study with published examples. JOURNAL OF CLINICAL EPIDEMIOLOGY. 2023;153:13–25.

1. Boutron 2020a

Boutron I, Chaimani A, Devane D, Meerpohl JJ, Rada G, Hróbjartsson A, et al. Interventions for the prevention and treatment of COVID‐19: a living mapping of research and living network meta‐analysis. Cochrane Database of Systematic Reviews [Internet]. 2020; Available from: <http://dx.doi.org/10.1002/14651858.CD013769>

1. Boutron 2020b

Boutron I, Chaimani A, Devane D, Meerpohl JJ, Rada G, Hróbjartsson A, et al. Interventions for the treatment of COVID‐19: a living network meta‐analysis. Cochrane Database of Systematic Reviews [Internet]. 2020; Available from: <http://dx.doi.org/10.1002/14651858.CD013770>

1. Burgard 2023

Burgard T, Studtrucker R, Bosnjak M. Living network meta-analyses for psychology in PsychOpen CAMA. INTERNATIONAL JOURNAL OF PSYCHOLOGY. 2023;58:745–745.

1. Brazinova 2018

Brazinova A, Rehorcikova V, Taylor MS, Buckova V, Majdan M, Psota M, et al. Epidemiology of Traumatic Brain Injury in Europe: A Living Systematic Review. Journal of neurotrauma [Internet]. 2018; Available from: <http://ovidsp.ovid.com/ovidweb.cgi?T=JS&PAGE=reference&D=emed19&NEWS=N&AN=628107540>

1. Breman 1992

Breman G, Albrecht B, Dale M, Hertel E. The living map: Bridging the gap. Bridges to Understanding. 1992;216–9.

1. Broekgaarden 2021

Broekgaarden F, Mandel I. Data set from: Rates of Compact Object Coalescences [Internet]. Zenodo. 2021. Available from: <https://zenodo.org/record/5847743/files/COC_rates_supplementary_material%20.pdf>

1. Brummer 2021

Brummer LE, Katzenschlager S, Gaeddert M, Erdmann C, Schmitz S, Bota M. Accuracy of novel antigen rapid diagnostics for SARS-CoV-2: A living systematic review and meta-analysis (vol 18, e1003735, 2021). PLOS MEDICINE. 2021;18.

1. Buitrago-Garcia 2020

Buitrago-Garcia D, Egli-Gany D, Counotte MJ, Hossmann S, Imeri H, Ipekci AM, et al. Data used in the publication: Occurrence and transmission potential of asymptomatic and pre-symptomatic SARS-CoV-2 infections: living systematic review and meta-analysis. Harvard Dataverse. 2020;2.

1. Bwire 2021

Bwire GM, Njiro BJ, Mwakawanga DL, Sabas D, Sunguya BF. Possible vertical transmission and antibodies against SARS-CoV-2 among infants born to mothers with COVID-19: A living systematic review. Journal of medical virology. 2021;93:1361–9.

1. Cabanac 2021

Cabanac G, Oikonomidi T, Boutron I. Day-to-day discovery of preprint-publication links. Scientometrics. 2021;1–20.

1. Cara 2022

Cara K.C., Beauchesne A.R., Li R., Chung M. Cochrane Review Summary on “Vitamin D Supplementation for the Treatment of COVID-19: A Living Systematic Review.” Journal of dietary supplements. 2022;19:143–5.

1. Carrasco 2020

Carrasco G, Morillas J, Calizaya M, Baeza I, Molina R, Meije Y. [ICU decision making based on Living Systematic Review strategy during SARS-CoV-2 pandemic. Results of a prospective case serie]. Decisiones en UCI basadas en la estrategia Living Systematic Review durante la pandemia de SARS-CoV-2 Resultados de una serie prospectiva de casos. 2020;44:516–9.

1. Centeno-Tablante 2021

Centeno-Tablante E, Medina-Rivera M, Finkelstein JL, Rayco-Solon P, Garcia-Casal MN, Rogers L, et al. Transmission of SARS-CoV-2 through breast milk and breastfeeding: a living systematic review. Annals of the New York Academy of Sciences. 2021;1484:32–54.

1. Chagla 2024

Chagla Z. In outpatients with COVID-19 during Omicron variant circulation, molnupiravir and nirmatrelvir-ritonavir improved outcomes. ANNALS OF INTERNAL MEDICINE. 2024;177:JC7–JC7.

1. Chai 2020

Chai KL, Valk SJ, Piechotta V, Kimber C, Monsef I, Doree C, et al. Convalescent plasma or hyperimmune immunoglobulin for people with COVID-19: a living systematic review. The Cochrane database of systematic reviews. 2020;10:CD013600.

Piechotta V, Iannizzi C, Chai KL, Valk SJ, Kimber C, Dorando E, et al. Convalescent plasma or hyperimmune immunoglobulin for people with COVID‐19: a living systematic review. Cochrane Database of Systematic Reviews [Internet]. 2021; Available from: http://dx.doi.org/10.1002/14651858.CD013600.pub4

Piechotta V, Chai KL, Valk SJ, Kimber C, Monsef I, Doree C, et al. ROB 2 assessments_Convalescent plasma or hyperimmune immunoglobulin for people with COVID-19_a living systematic review. Zenodo. 2020;

Piechotta V, Chai KL, Valk SJ, Doree C, Monsef I, Wood EM, et al. Convalescent plasma or hyperimmune immunoglobulin for people with COVID-19: a living systematic review. The Cochrane database of systematic reviews. 2020;7:CD013600.

Piechotta V, Iannizzi C, Chai KL, Valk SJ, Kimber C, Dorando E, et al. Risk of bias assessments for version 4 of the Cochrane review “Convalescent plasma or hyperimmune immunoglobulin for people with COVID-19: a living systematic review.” Zenodo. 2021;

Piechotta V, Monsef I, Chai KL, Wood EM, McQuilten Z, Doree C, et al. Convalescent plasma or hyperimmune immunoglobulin for people with COVID-19: a rapid review. Cochrane Database of Systematic Reviews. 2020;2020:CD013600.

Piechotta V, Monsef I, Kimber C, Doree C, Roberts DJ, Chai KL, et al. Convalescent plasma and hyperimmune immunoglobulin to prevent infection with SARS-CoV-2. Cochrane Database of Systematic Reviews. 2021;2020:CD013802.

Iannizzi C, Chai K, Piechotta V, Valk S, Kimber C, Monsef I, et al. Convalescent plasma for people with COVID‐19: a living systematic review. Cochrane Database of Systematic Reviews [Internet]. 2023; Available from: http://dx.doi.org/10.1002/14651858.CD013600.pub6

1. Challoumas 2023

Challoumas D, Crosbie G, O’Neill S, Pedret C, Millar N. Effectiveness of Exercise Treatments with or without Adjuncts for Common Lower Limb Tendinopathies: A Living Systematic Review and Network Meta-analysis. SPORTS MEDICINE-OPEN. 2023;9.

1. Charide 2023

Charide R., Stallwood L., Munan M., Sayfi S., Hartling L., Butcher N.J., et al. Knowledge mobilization activities to support decision-making by youth, parents, and adults using a systematic and living map of evidence and recommendations on COVID-19: protocol for three randomized controlled trials and qualitative user-experience studi. medRxiv [Internet]. 2022; Available from: <https://www.medrxiv.org/>

Charide R, Stallwood L, Munan M, Sayfi S, Hartling L, Butcher N, et al. Knowledge mobilization activities to support decision-making by youth, parents, and adults using a systematic and living map of evidence and recommendations on COVID-19: protocol for three randomized controlled trials and qualitative user-experience studi. TRIALS. 2023;24.

1. Chen 2024

Chen J, Jin Z, Luo B, Wang Y, Li R, Zhu H, et al. New First-line Immunotherapy-based Therapies for Unresectable Hepatocellular Carcinoma: A Living Network Meta-analysis. JOURNAL OF CLINICAL AND TRANSLATIONAL HEPATOLOGY. 2024;12:15–24.

1. Choi 2023

Choi M, Lee H, Yu S, Kim J, Park J, Ryoo S, et al. Two Years of Experience and Methodology of Korean COVID-19 Living Clinical Practice Guideline Development. JOURNAL OF KOREAN MEDICAL SCIENCE. 2023;38.

1. Chou 2020a

Chou R, Dana T, Jungbauer R, Weeks C, McDonagh MS. Masks for Prevention of Respiratory Virus Infections, Including SARS-CoV-2, in Health Care and Community Settings : A Living Rapid Review. Annals of internal medicine. 2020;173:542–55.

Anonymous. Erratum: Update Alert 8: Masks for Prevention of Respiratory Virus Infections, Including SARS-CoV-2, in Health Care and Community Settings (Ann Intern Med. (2022) 175 (W108-W109) DOI: 10.7326/L22-0272). Annals of Internal Medicine. 2022;175:1627.

Chou R, Dana T. Major Update: Masks for Prevention of SARS-CoV-2 in Health Care and Community Settings-Final Update of a Living, Rapid Review. ANNALS OF INTERNAL MEDICINE. 2023;176:827-+.

1. Chou 2020b

Chou R, Dana T, Buckley DI, Selph S, Fu R, Totten AM. Epidemiology of and Risk Factors for Coronavirus Infection in Health Care Workers: A Living Rapid Review. Annals of internal medicine. 2020;173:120–36.

1. Chou 2021

Chou R. Living and Rapid Reviews for Covid-19 to Inform Guidance and Policy. Annals of Behavioral Medicine. 2021;55:S612–S612.

1. Ciapponi 2023

Ciapponi A, Berrueta M, Ballivian J, Bardach A, Mazzoni A, Anderson S, et al. Safety, immunogenicity, and effectiveness of COVID-19 vaccines for pregnant persons: A protocol for systematic review and meta analysis. MEDICINE. 2023;102.

1. Cnossen 2021

Cnossen MC, Scholten AC, Lingsma HF, Synnot A, Tavender E, Gantner D, et al. Adherence to Guidelines in Adult Patients with Traumatic Brain Injury: A Living Systematic Review. Journal of neurotrauma. 2021;38:1072–85.

1. Codd 2021

Codd AS, Hanna SJ, Compeer EB, Richter FC, Pring EJ, Gea-Mallorqui E, et al. Neutrophilia, lymphopenia and myeloid dysfunction: a living review of the quantitative changes to innate and adaptive immune cells which define COVID-19 pathology. Oxford open immunology. 2021;2:iqab016.

1. Cohen 2021

Cohen MA, Paynter R, Edelman A, Stewart F, Henderson J. Risk of thromboembolism in patients with COVID-19 who are using hormonal contraception. Cochrane Database of Systematic Reviews. 2021;2021:CD014908.

1. COOPConsortium 2024

COOP Consortium, Zahra A, van Smeden M, Abbink E, van den Berg J, Blom M, et al. External validation of six COVID-19 prognostic models for predicting mortality risk in older populations in a hospital, primary care, and nursing home setting. JOURNAL OF CLINICAL EPIDEMIOLOGY. 2024;168.

1. Cooper 2023

Cooper N, Germeni E, Freeman S, Jaiswal N, Nevill C, Sutton A, et al. New horizons in evidence synthesis for older adults. AGE AND AGEING. 2023;52.

1. Costa 2024

Costa J, Pothier J, Bosis E, Boch J, Kölliker R, Koebnik R. A Community-Curated DokuWiki Resource on Diagnostics, Diversity, Pathogenicity, and Genetic Control of Xanthomonads. MOLECULAR PLANT-MICROBE INTERACTIONS. 2024;37:347–53.

1. Counotte 2017

Counotte MJ, Low N, Maxwell L, Kim CR, Broutet NJN. Sexual transmission of flaviviruses-a living systematic review. Sexually transmitted infections. 2017;93:A33–4.

1. Counette 2019

Counotte MJ. Living systematic review on adverse outcomes of Zika - Figures and Table. Harvard Dataverse. 2019;3.

Counotte MJ. Living systematic review on adverse outcomes of Zika - Supplementary Material. Harvard Dataverse. 2019;1.

1. Cruciani 2021

Cruciani F, Amato L, De Crescenzo F, Mitrova Z, Saulle R, Vecchi S, et al. [The praise of uncertainty: a systematic living review to evaluate the efficacy and safety of drug treatments for patients with covid-19.]. L’elogio dell’incertezza: una revisione sistematica living per valutare l’efficacia e la sicurezza dei trattamenti farmacologici per pazienti affetti da covid-19. 2021;112:195–206.

1. Currie 2020

Currie G, Macleod M, Sena E, Bahor Z, Liao J, Sena C, et al. A “Living” Evidence Summary of Primary Research Related to Covid-19. Open Science Framework. 2020;

Currie G, Macleod M, Sena E, Bahor Z, Liao J, Sena C, et al. Protocol for a “Living” Evidence Summary of Primary Research Related to Covid-19. Open Science Framework. 2020;

1. Cuijpers 2023

Cuijpers P, Harrer M, Miguel C, Ciharova M, Karyotaki E. Five Decades of Research on Psychological Treatments of Depression: A Historical and Meta-Analytic Overview. AMERICAN PSYCHOLOGIST. 2023;

1. Dahal 2021

Dahal P., Singh-Phulgenda S., Ngu R., Rashan S., Brack M., Maguire B., et al. Haemoglobin at inclusion in visceral leishmaniasis clinical studies: A systematic review and proposal for an individual patient data meta-analysis. Tropical Medicine and International Health. 2021;26:55–6.

1. da Rocha 2020

da Rocha AP, Atallah AN, Pereira Nunes Pinto AC, Rocha-Filho CR, Milby KM, Civile VT, et al. COVID-19 and patients with immune-mediated inflammatory diseases undergoing pharmacological treatments: a rapid living systematic review. Sao Paulo Medical Journal. 2020;138:515–29.

1. Davidson 2022

Davidson M, Menon S, Chaimani A, Evrenoglou T, Ghosn L, Graña C, et al. Interleukin‐1 blocking agents for treating COVID‐19. Cochrane Database of Systematic Reviews [Internet]. 2022; Available from: <http://dx.doi.org/10.1002/14651858.CD015308>

1. Davidson 2024

Davidson M, Evrenoglou T, Grana C, Chaimani A, Boutron I. Comparison of effect estimates between preprints and peer-reviewed journal articles of COVID-19 trials. BMC MEDICAL RESEARCH METHODOLOGY. 2024;24.

1. Davies 2019

Davies C, De Micheli A, Brondino N, Fusar-Poli P, Solmi M, Kotlicka-Antczak M, et al. Preventive Treatments for Psychosis: Umbrella Review (Just the Evidence). Frontiers in psychiatry. 2019;10:764.

1. Dawit 2022

Dawit H, Absi M, Islam N, Ebrahimzadeh S, McInnes MDF. Diagnostic accuracy of thoracic imaging modalities for the detection of COVID-19. World journal of radiology. 2022;14:47–9.

1. deJong 2022

de Jong VMT, Rousset RZ, Antonio-Villa NE, Buenen AG, Van Calster B, Bello-Chavolla OY, et al. Clinical prediction models for mortality in patients with covid-19: external validation and individual participant data meta-analysis. BMJ (Clinical research ed). 2022;378:e069881.

1. deOliveira 2023

de Oliveira A, Galante M, Maia L, Craveiro I, da Silva A, Fronteira I, et al. Policies and Management Interventions to Enhance Health and Care Workforce Capacity for Addressing the COVID-19 Pandemic: Protocol for a Living Systematic Review. JMIR RESEARCH PROTOCOLS. 2023;12.

1. Decary 2021

Decary S., Dugas M., Stefan T., Langlois L., Skidmore B., Bhereur A., et al. Care models for long COVID: A rapid systematic review. medRxiv [Internet]. 2021; Available from: <https://www.medrxiv.org>

1. Deeks 2020

Deeks JJ, Dinnes J, Takwoingi Y, Davenport C, Spijker R, Taylor-Phillips S, et al. Antibody tests for identification of current and past infection with SARS‐CoV‐2. Cochrane Database of Systematic Reviews [Internet]. 2020; Available from: <http://dx.doi.org/10.1002/14651858.CD013652>

1. Desimmie 2021

Desimmie B.A., Raru Y.Y., Awadh H.M., He P., Teka S., Willenburg K.S. Insights into sars-cov-2 persistence and its relevance. Viruses. 2021;13:1025.

1. Dinnes 2020

Deeks JJ, Taylor M, Adriano A, Davenport C, Dittrich S, Emperador D, et al. Rapid, point-of-care antigen and molecular-based tests for diagnosis of SARS-CoV-2 infection. Cochrane Database of Systematic Reviews. 2021;2021:CD013705.

Dinnes J, Deeks JJ, Adriano A, Berhane S, Davenport C, Dittrich S, et al. Rapid, point-of-care antigen and molecular-based tests for diagnosis of SARS-CoV-2 infection. The Cochrane database of systematic reviews. 2020;8:CD013705.

Dinnes J, Deeks JJ, Berhane S, Taylor M, Adriano A, Davenport C, et al. Rapid, point-of-care antigen and molecular-based tests for diagnosis of SARS-CoV-2 infection. The Cochrane database of systematic reviews. 2021;3:CD013705.

Dinnes J., Sharma P., Berhane S., van Wyk S.S., Nyaaba N., Domen J., et al. Rapid, point-of-care antigen tests for diagnosis of SARS-CoV-2 infection. Cochrane Database of Systematic Reviews. 2022;2022:CD013705.

1. Discepola 2021

Discepola M-N, Carboni-Jimenez A, Kwakkenbos L, Henry RS, Boruff J, Krishnan A, et al. Effects of non-pharmacological and non-surgical interventions on health outcomes in systemic sclerosis: protocol for a living systematic review. BMJ open. 2021;11:e047428.

1. Dixit 2021

Dixit A., Zhou D., Sheikh J., Lawson H., Kew T., Ansari K., et al. Prevalence and risk-factors of COVID-19 in pregnancy: Living systematic review and metaanalysis. BJOG: An International Journal of Obstetrics and Gynaecology. 2021;128:196–7.

1. Domingo 2021

Domingo F.R., Waddell L.A., Cheung A.M., Cooper C.L., Belcourt V.J., Zuckermann A.M.E., et al. Prevalence of long-term effects in individuals diagnosed with COVID-19: an updated living systematic review. medRxiv [Internet]. 2021; Available from: <https://www.medrxiv.org/>

1. Dressler 2019

Dressler C, Zidane M, Grine L, Galdas P, Lambert J, Nast A. Therapeutic patient education and self-management support for patients with psoriasis - a living systematic review and meta-analysis. Journal of the European Academy of Dermatology and Venereology. 2019;33:8–8.

1. Drucker 2018

Drucker AM, Ellis A, Jabbar-Lopez Z, Flohr C, Yiu ZZN, Arents BWM, et al. Systemic immunomodulatory treatments for atopic dermatitis: Protocol for a systematic review with network meta-analysis. BMJ open. 2018;8:e023061.

Drucker AM, Ellis AG, Bohdanowicz M, Mashayekhi S, Yiu ZZN, Rochwerg B, et al. Systemic Immunomodulatory Treatments for Atopic Dermatitis: A Living Systematic Review and Network Meta-Analysis. Acta Dermato-Venereologica. 2021;101:28–28.

Drucker A.M., Morra D.E., Prieto-Merino D., Ellis A.G., Yiu Z.Z.N., Rochwerg B., et al. Systemic Immunomodulatory Treatments for Atopic Dermatitis: Update of a Living Systematic Review and Network Meta-analysis. JAMA Dermatology. 2022;158:523–32.

Morra D.E., Ellis A.G., Mashayekhi S., Yiu Z.Z., Rochwerg B., Giorgio S.D., et al. Systemic Immunomodulatory Treatments for Patients with Atopic Dermatitis - Update of a Living Network Meta-analysis. Journal of Cutaneous Medicine and Surgery. 2021;25:29S-30S.

Drucker A, Lam M, Elsawi R, Prieto-Merino D, Malek R, Ellis A, et al. Comparing binary efficacy outcomes for systemic immunomodulatory treatments for atopic dermatitis in a living systematic review and network meta-analysis. BRITISH JOURNAL OF DERMATOLOGY. 2023;

1. Duan 2022

Duan Y., Luo J., Zhao L., Zhang X., Miao J., Moher D., et al. Reporting and data sharing level for COVID-19 vaccine trials: A cross-sectional study. eBioMedicine. 2022;78:103962.

1. Duarte 2020

Duarte G, Ortiz-Munoz L, Belen Morales M, Paz Acuna M, Rada G. Sexual transmission of SARS-CoV-2 virus and its role in the spread of COVID-19: A living systematic review protocol. Medwave. 2020;20.

1. Dumitrascu 2021

Dumitrascu F, Branje KE, Hladkowicz ES, Lalu M, McIsaac DI. Association of frailty with outcomes in individuals with COVID-19: A living review and meta-analysis. Journal of the American Geriatrics Society. 2021;

1. Ebbesen 2023

Ebbesen B, Modrau B, Kontou E, Finch E, Crowfoot G, Crow J, et al. Lasting impairments following transient ischemic attack and minor stroke: a systematic review protocol. FRONTIERS IN NEUROLOGY. 2023;14.

1. Edgar 2024

Edgar K, Iliffe S, Doll H, Clarke M, Gonçalves-Bradley D, Wong E, et al. Admission avoidance hospital at home. Cochrane Database of Systematic Reviews [Internet]. 2024; Available from: http://dx.doi.org/10.1002/14651858.CD007491.pub3

1. Egunsola 2022a

Egunsola O, Verhoek A, Liu J, Thorlund K, Heeg B, Kwon C, et al. LIVING NETWORK META-ANALYSIS FOR UP-TO-DATE COMPARATIVE EFFECTIVENESS: A CASE STUDY IN MULTIPLE MYELOMA MAINTENANCE. VALUE IN HEALTH. 2022;25:S325–S325.

1. Egunsola 2022b

Egunsola O, Verhoek A, Liu J, Thorlund K, Heeg B, Kwon C, et al. LIVING NETWORK META-ANALYSIS FOR UP-TO-DATE COMPARATIVE EFFECTIVENESS: A CASE STUDY IN METASTATIC CASTRATION RESISTANT PROSTATE CANCER (MCRPC). VALUE IN HEALTH. 2023;26:S265–S265.

1. Elangovan 2022

Elangovan S. TASTE DISORDERS AND XEROSTOMIA ARE HIGHLY PREVALENT IN PATIENTS WITH COVID-19. The journal of evidence-based dental practice. 2022;22:101687.

1. ElMikati 2023

El Mikati I, Morgan R, Murad M, Sultan S, Falck-Ytter Y, Mustafa R. Testing guidelines during times of crisis: challenges and limitations of developing rapid and living guidelines. CLINICAL MICROBIOLOGY AND INFECTION. 2023;29:424–8.

1. Elsabbagh 2022

Elsabbagh M., Yusuf A., Zeidan J., Scorah J., Fombonne E., Durkin M.S., et al. The time has come for living systematic reviews in autism research. Autism Research. 2022;15:1187–8.

1. Elvidge 2022

Elvidge J, Summerfield A, Nicholls D, Dawoud D. TREATMENTS AND DIAGNOSTICS FOR COVID-19: A LIVING SYSTEMATIC REVIEW OF ECONOMIC EVALUATIONS. VALUE IN HEALTH. 2022;25:S423–S423.

Elvidge J, Hopkin G, Narayanan N, Nicholls D, Dawoud D. Diagnostics and treatments of COVID-19: two-year update to a living systematic review of economic evaluations. FRONTIERS IN PHARMACOLOGY. 2023;14.

1. Elviro 2023

Elviro C, Longcroft-Harris B, Allin E, Leache L, Woo K, Bone J, et al. Late Breaking Abstract - A living network meta-analysis comparing modalities in the management of pediatric empyema. EUROPEAN RESPIRATORY JOURNAL. 2023;62.

1. Estcourt 2022

Estcourt LJ, Cohn CS, Pagano MB, Iannizzi C, Kreuzberger N, Skoetz N, et al. Clinical Practice Guidelines From the Association for the Advancement of Blood and Biotherapies (AABB): COVID-19 Convalescent Plasma. Annals of internal medicine. 2022;175:1310–21.

1. EuropeanSocClinMicrobiolInfect 2023

European Soc Clin Microbiol Infect, Fragkou P, Moschopoulos C, Dimopoulou D, Ong D, Dimopoulou K, et al. Performance of point-of care molecular and antigen-based tests for SARS-CoV-2: a living systematic review and meta-analysis. CLINICAL MICROBIOLOGY AND INFECTION. 2023;29:291–301.

1. Farina 2017

Farina N, Llewellyn D, Isaac M, Tabet N. Vitamin E for Alzheimer’s dementia and mild cognitive impairment. Cochrane Database of Systematic Reviews [Internet]. 2017; Available from: <http://dx.doi.org/10.1002/14651858.CD002854.pub5>

1. Fernando 2021

Fernando SM, Rochwerg B. In COVID-19, tocilizumab reduces all-cause mortality at 28 d. Annals of internal medicine. 2021;174:JC63.

1. Ferrara 2021

Ferrara R, Imbimbo M, Malouf R, Calais F, Marchal C, Westeel V, et al. Single or combined immune checkpoint inhibitors compared to first-line platinum-based chemotherapy with or without bevacizumab for people with advanced non-small cell lung cancer. Cochrane Database of Systematic Reviews. 2020;2020:CD013257.

Ferrara R, Imbimbo M, Malouf R, Paget-Bailly S, Calais F, Marchal C, et al. Single or combined immune checkpoint inhibitors compared to first-line platinum-based chemotherapy with or without bevacizumab for people with advanced non-small cell lung cancer. The Cochrane database of systematic reviews. 2021;4:CD013257.

Ferrara R, Imbimbo M, Paget-Bailly S, Malouf R, Calais F, Agazzi GM, et al. Single or combined immune checkpoint inhibitors compared to first-line chemotherapy with or without bevacizumab for people with advanced non-small cell lung cancer. Cochrane Database of Systematic Reviews. 2019;2019:CD013257.

1. Fragkou 2022

Fragkou P.C., Palaiodimou L., Stefanou M.I., Katsanos A.H., Lambadiari V., Paraskevis D., et al. Effects of low molecular weight heparin and fondaparinux on mortality, hemorrhagic and thrombotic complications in COVID-19 patients. Therapeutic Advances in Neurological Disorders [Internet]. 2022;15. Available from: <https://journals.sagepub.com/home/TAN>

Fragkou. EFFECTS OF LOW MOLECULAR WEIGHT HEPARIN AND FONDAPARINUX ON MORTALITY, HEMORRHAGIC AND THROMBOTIC COMPLICATIONS IN COVID-19 PATIENTS: A SYSTEMATIC REVIEW AND META-ANALYSIS. ESOC 2022 Abstract Book. European Stroke Journal. 2022;7:3–545.

1. Fusar-Poli 2019

Fusar-Poli P, Davies C, Solmi M, Brondino N, De Micheli A, Kotlicka-Antczak M, et al. DataSheet_1_Preventive Treatments for Psychosis: Umbrella Review (Just the Evidence).docx. Figshare. 2019;

1. Fronteira 2024

Fronteira I, Mathews V, dos Santos R, Matsumoto K, Amde W, Pereira A, et al. Impacts for health and care workers of Covid-19 and other public health emergencies of international concern: living systematic review, meta-analysis and policy recommendations. HUMAN RESOURCES FOR HEALTH. 2024;22.

1. Gasparini 2022

Gasparini L., Tsuji S., Bergmann C. Ten easy steps to conducting transparent, reproducible meta-analyses for infant researchers. Infancy. 2022;27:736–64.

1. Geisler 2020

Geisler BP, Zahabi L, Lang AE, Eastwood N, Tennant E, Lukic L, et al. Repurposing Existing Medications for Coronavirus Disease 2019: Protocol for a Rapid and Living Systematic Review. medRxiv : the preprint server for health sciences [Internet]. 2020; Available from: http://ovidsp.ovid.com/ovidweb.cgi?T=JS&PAGE=reference&D=pmnm&NEWS=N&AN=32511471

Geisler BP, Zahabi L, Lang AE, Eastwood N, Tennant E, Lukic L, et al. Repurposing existing medications for coronavirus disease 2019: protocol for a rapid and living systematic review. Systematic reviews. 2021;10:143.

1. Ghosn 2021

Ghosn L, Chaimani A, Evrenoglou T, Davidson M, Graña C, Schmucker C, et al. Interleukin‐6 blocking agents for treating COVID‐19: a living systematic review. Cochrane Database of Systematic Reviews [Internet]. 2021; Available from: <http://dx.doi.org/10.1002/14651858.CD013881>

Ghosn L, Assi R, Evrenoglou T, Buckley B, Henschke N, Probyn K, et al. Interleukin‐6 blocking agents for treating COVID‐19: a living systematic review. Cochrane Database of Systematic Reviews [Internet]. 2023; Available from: http://dx.doi.org/10.1002/14651858.CD013881.pub2

1. Giustini 2018

Giustini D, Ali SM, Fraser M, Kamel Boulos MN. Effective uses of social media in public health and medicine: a systematic review of systematic reviews. Online journal of public health informatics. 2018;10:e215.

1. Glennon 2021

Glennon V, Whittle S, Hill C, Johnston R, Avery J, Grobler L, et al. Short‐term glucocorticoids for flares in people with rheumatoid arthritis receiving disease‐modifying anti‐rheumatic drugs (DMARDs). Cochrane Database of Systematic Reviews [Internet]. 2021; Available from: <http://dx.doi.org/10.1002/14651858.CD014898>

1. Goldkhule 2020

Goldkuhle M, Aldin A, Jakob T, Adams A, Monsef I, Heidenreich A, et al. First‐line therapy for adults with advanced renal cell carcinoma: a systematic review and network meta‐analysis. Cochrane Database of Systematic Reviews [Internet]. 2020; Available from: <http://dx.doi.org/10.1002/14651858.CD013798>

Aldin A, Besiroglu B, Adams A, Monsef I, Piechotta V, Tomlinson E, et al. First‐line therapy for adults with advanced renal cell carcinoma: a systematic review and network meta‐analysis. Cochrane Database of Systematic Reviews [Internet]. 2023; Available from: http://dx.doi.org/10.1002/14651858.CD013798.pub2

1. Gomes 2020

Gomes JFC. Nursing knowledge on skin ulcers healing - a living scoping review protocol. Open Science Framework. 2020;

1. Gomez-Ochoa 2021

Gomez-Ochoa SA, Franco OH, Rojas LZ, Raguindin PF, Roa-Diaz ZM, Wyssmann BM, et al. COVID-19 in Health-Care Workers: A Living Systematic Review and Meta-Analysis of Prevalence, Risk Factors, Clinical Characteristics, and Outcomes. American journal of epidemiology. 2021;190:161–75.

1. Graña 2022

Graña C, Ghosn L, Evrenoglou T, Jarde A, Minozzi S, Bergman H, et al. Efficacy and safety of COVID‐19 vaccines. Cochrane Database of Systematic Reviews [Internet]. 2022; Available from: http://dx.doi.org/10.1002/14651858.CD015477

1. Griesel 2022

Griesel M, Wagner C, Mikolajewska A, Stegemann M, Fichtner F, Metzendorf M-I, et al. Inhaled corticosteroids for the treatment of COVID‐19. Cochrane Database of Systematic Reviews [Internet]. 2022; Available from: <http://dx.doi.org/10.1002/14651858.CD015125>

1. Guerin 2021

Guerin P. All associated data including underlying and extended data and supplementary materials for the publication: McLean et al. The fragmented COVID-19 therapeutics research landscape: a living systematic review of clinical trial registrations evaluating priori. Harvard Dataverse. 2021.

1. Guleid 2020

Guleid F, Holtman GA, Yang B, Langendam M, Leeflang MMG, Dittrich S, et al. Routine laboratory testing to determine if a patient has COVID-19. Cochrane Database of Systematic Reviews. 2020;2020:CD013787.

1. Hanna 2021

Hanna SJ, Codd AS, Gea-Mallorqui E, Scourfield DO, Richter FC, Ladell K, et al. T cell phenotypes in COVID-19 - a living review. Oxford open immunology. 2021;2:iqaa007.

1. Harder 2021

Harder T., Koch J., Vygen-Bonnet S., Kulper-Schiek W., Pilic A., Reda S., et al. Efficacy and effectiveness of COVID-19 vaccines against SARS-CoV-2 infection: interim results of a living systematic review, 1 January to 14 May 2021. Euro surveillance : bulletin Europeen sur les maladies transmissibles = European communicable disease bulletin. 2021;26.

Harder T., Kulper-Schiek W., Reda S., Treskova-Schwarzbach M., Koch J., Vygen-Bonnet S., et al. Effectiveness of COVID-19 vaccines against SARSCoV-2 infection with the Delta (B.1.617.2) variant: second interim results of a living systematic review and meta-analysis, 1 January to 25 August 2021. Eurosurveillance [Internet]. 2021;26. Available from: <https://www.eurosurveillance.org/content/10.2807/1560-7917.ES.2021.26.41.2100920>

1. Hartmann-Boyce 2022

Hartmann-Boyce J, Lindson N, Butler AR, McRobbie H, Bullen C, Begh R, et al. Electronic cigarettes for smoking cessation. The Cochrane database of systematic reviews. 2022;11:CD010216.

1. Harrrington 2017

Harrington L. Closing the Science-Health Gap With Technology: Evidence-Based Living. AACN advanced critical care. 2017;28:102–6.

1. Harris 2023

Harris E. “Living” Systematic Review of Mental Health During Pandemic. JAMA-JOURNAL OF THE AMERICAN MEDICAL ASSOCIATION. 2023;329:1054–1054.

1. Hawthrone 2021

Hawthorne G., Harvey A. Field clinical performance of SARS-CoV-2 point-of-care diagnostic tests: A living systematic review of trials up to 17th of August, 2021. medRxiv [Internet]. 2021; Available from: <https://www.medrxiv.org/>

1. Hawton 2015

Hawton K, Witt KG, Taylor Salisbury TL, Arensman E, Gunnell D, Townsend E, et al. Interventions for self-harm in children and adolescents. Cochrane Database of Systematic Reviews. 2015;

1. Helfand 2022

Helfand M., Fiordalisi C., Wiedrick J., Ramsey K.L., Armstrong C., Gean E., et al. Risk for Reinfection after SARS-CoV-2: A Living, Rapid Review for American College of Physicians Practice Points on the Role of the Antibody Response in Conferring Immunity following SARS-CoV-2 Infection. Annals of Internal Medicine. 2022;175:547–55.

1. Hernandez 2020

Hernandez AV, Roman YM, Pasupuleti V, Barboza JJ, White CM. Hydroxychloroquine or Chloroquine for Treatment or Prophylaxis of COVID-19: A Living Systematic Review. Annals of internal medicine. 2020;173:287–96.

Hernandez AV, Roman YM, Pasupuleti V, Barboza JJ, White CM. Update Alert: Hydroxychloroquine or Chloroquine for the Treatment or Prophylaxis of COVID-19. Annals of Internal Medicine. 2020;173:78–9.

Hernandez AV, Roman YM, Pasupuleti V, Barboza JJ, White CM. Update Alert 2: Hydroxychloroquine or Chloroquine for the Treatment or Prophylaxis of COVID-19. Annals of Internal Medicine. 2020;173:W128–9.

Hernandez AV, Roman YM, Pasupuleti V, Barboza JJ, White CM. Update Alert 3: Hydroxychloroquine or Chloroquine for the Treatment or Prophylaxis of COVID-19. Annals of Internal Medicine. 2020;173:W156–7.

1. Hernandez-Vasquez 2022

Hernandez-Vasquez A., Barrenechea-Pulache A., Comande D., Azanedo D. Mouthrinses and SARS-CoV-2 viral load in saliva: a living systematic review. Evidence-based dentistry [Internet]. 2022; Available from: <https://www.nature.com/articles/s41432-022-0253-z.pdf>

1. Hirsch 2021

Hirsch C, Valk SJ, Piechotta V, Chai KL, Estcourt LJ, Monsef I, et al. SARS‐CoV‐2‐neutralising monoclonal antibodies to prevent COVID‐19. Cochrane Database of Systematic Reviews [Internet]. 2021; Available from: <http://dx.doi.org/10.1002/14651858.CD014945>

Hirsch C, Park Y, Piechotta V, Chai K, Estcourt L, Monsef I, et al. SARS‐CoV‐2‐neutralising monoclonal antibodies to prevent COVID‐19. Cochrane Database of Systematic Reviews [Internet]. 2022; Available from: <http://dx.doi.org/10.1002/14651858.CD014945.pub2>

1. Hirt 2022

Hirt J, Janiaud P, Hemkens LG. Clinical trial research agenda on COVID-19 - the first two years in Germany and beyond. Zeitschrift fur Evidenz, Fortbildung und Qualitat im Gesundheitswesen. 2022;174:32–42.

1. Hirt 2023

Hirt J, Adlbrecht L, Maurer C, Beer T. Exploring experiences of times without care and encounters in dementia: protocol for a living and adaptive evidence map. BMJ OPEN. 2023;13.

1. Hodder 2017

Hodder RK, Stacey FG, Wyse RJ, O’Brien KM, Clinton-McHarg T, Tzelepis F, et al. Interventions for increasing fruit and vegetable consumption in children aged five years and under. The Cochrane database of systematic reviews. 2017;9:CD008552.

1. Holmer 2022

Holmer HK, Mackey K, Fiordalisi CV, Armstrong C, Gean E, Arkhipova-Jenkins I, et al. Antibody Response Following SARS-CoV-2 Infection and Implications for Immunity: Final Update of a Rapid, Living Review. 2022;

1. Holmer 2023

Holmer H, Mackey K, Fiordalisi C, Helfand M. Major Update 2: Antibody Response and Risk for Reinfection After SARS-CoV-2 Infection-Final Update of a Living, Rapid Review. ANNALS OF INTERNAL MEDICINE. 2023;176:85-+.

1. Hua 2020

Hua C, Mazaud C, Sbidian E, Le Cleach L, Chaimani A, Afach S, et al. Systemic pharmacological treatments for chronic plaque psoriasis: a network meta-analysis. Cochrane Database of Systematic Reviews. 2020;2020:CD011535.

Sbidian E, Chaimani A, Guelimi R, Garcia-Doval I, Hua C, Hughes C, et al. Systemic pharmacological treatments for chronic plaque psoriasis: a network meta‐analysis. Cochrane Database of Systematic Reviews [Internet]. 2023; Available from: http://dx.doi.org/10.1002/14651858.CD011535.pub6

1. Hudda 2022

Hudda M.T., Archer L., van Smeden M., Moons K.G.M., Collins G.S., Steyerberg E.W., et al. Minimal reporting improvement after peer review in reports of covid-19 prediction models: systematic review. Journal of clinical epidemiology. 2022;

Hudda M, Archer L, van Smeden M, Moons K, Collins G, Steyerberg E, et al. Minimal reporting improvement after peer review in reports of COVID-19 prediction models: systematic review. JOURNAL OF CLINICAL EPIDEMIOLOGY. 2023;154:75–84.

1. Hunter 2021

Hunter J., Arentz S., Yang G., Goldenberg J., Myers S.P., Mertz D., et al. Zinc for the prevention and treatment of SARS-CoV-2 and other acute viral respiratory infections - a living rapid review and meta-analysis. European Journal of Integrative Medicine. 2021;48:101902.

1. Hussain 2021

Hussain S, Riad A, Singh A, Klugarova J, Antony B, Banna H, et al. Global Prevalence of COVID-19-Associated Mucormycosis (CAM): Living Systematic Review and Meta-Analysis. Journal of fungi (Basel, Switzerland). 2021;7.

1. Ianizzi 2021

Iannizzi C., Akl E.A., Kahale L.A., Dorando E., Mosunmola Aminat A., Barker J.M., et al. Methods and guidance on conducting, reporting, publishing and appraising living systematic reviews: A scoping review protocol. F1000Research. 2021;10:802.

1. Islam 2020

Islam N, Salameh J-P, Leeflang MM, Hooft L, McGrath TA, van der Pol CB, et al. Thoracic imaging tests for the diagnosis of COVID-19. The Cochrane database of systematic reviews. 2020;11:CD013639.

Salameh J-P, Leeflang MM, Hooft L, Islam N, McGrath TA, van der Pol CB, et al. Thoracic imaging tests for the diagnosis of COVID-19. The Cochrane database of systematic reviews. 2020;9:CD013639.

Islam N, Ebrahimzadeh S, Salameh J-P, Kazi S, Fabiano N, Treanor L, et al. Thoracic imaging tests for the diagnosis of COVID-19. The Cochrane database of systematic reviews. 2021;3:CD013639.

Ebrahimzadeh S, Islam N, Dawit H, Salameh J-P, Kazi S, Fabiano N, et al. Thoracic imaging tests for the diagnosis of COVID‐19. Cochrane Database of Systematic Reviews [Internet]. 2022; Available from: <http://dx.doi.org/10.1002/14651858.CD013639.pub5>

1. INTER-ChangeTeam 2023

INTER-Change Team, Fortin A, Lavoie K, Ben-Porat T, Yousefi R, Peláez S, et al. Coconstruction of Adjunct Behavioral Interventions to Bariatric Surgery: The INTER-Change Program. HEALTH PSYCHOLOGY. 2023;42:343–52.

1. Jahan 2022

Jahan N., Brahma A., Kumar M.S., Bagepally B.S., Ponnaiah M., Bhatnagar T., et al. Corrigendum to “Seroprevalence of IgG antibodies against SARS-CoV-2 in India, March 2020-August 2021: a systematic review and meta-analysis” [International Journal of Infectious Diseases, Volume 116 (2022) P59-67](S1201971221012510)(10.1016/j.ijid.2021.12. International Journal of Infectious Diseases. 2022;119:119.

1. Jammu 2021

Jammu AS, Chasen MR, Lofters AK, Bhargava R. Systematic rapid living review of the impact of the COVID-19 pandemic on cancer survivors: update to August 27, 2020. Supportive care in cancer : official journal of the Multinational Association of Supportive Care in Cancer. 2021;29:2841–50.

1. Janiaud 2021

Janiaud P., Hemkens L.G., Ioannidis J.P.A. Challenges and Lessons Learned From COVID-19 Trials: Should We Be Doing Clinical Trials Differently? Canadian Journal of Cardiology. 2021;37:1353–64.

1. Jiang 2022

Jiang L, Tang K, Irfan O, Li X, Zhang E, Bhutta Z. Epidemiology, Clinical Features, and Outcomes of Multisystem Inflammatory Syndrome in Children (MIS-C) and Adolescents-a Live Systematic Review and Meta-analysis. Current pediatrics reports. 2022;10:19–30.

1. Jimenez-Mora 2021

Jimenez-Mora M.A., Varela A.R., Meneses-Echavez J.F., Bidonde J., Angarita-Fonseca A., Siemieniuk R.A.C., et al. Patient-important outcomes reported in randomized controlled trials of pharmacologic treatments for COVID-19: a protocol of a META-epidemiological study. Systematic Reviews. 2021;10:289.

1. Kacimi 2021

Kacimi S.E.O., Greca E., Haireche M.A., ElHawary A.S., Ould Setti M., Caruana R., et al. The Place of Fluvoxamine in the Treatment of Non-critically ill Patients with COVID-19: A Living Systematic Review and Meta-analysis. medRxiv [Internet]. 2021; Available from: <https://www.medrxiv.org/>

1. Kahale 2017

Kahale LA, Hakoum MB, Tsolakian IG, Matar CF, Barba M, Yosuico VED, et al. Oral anticoagulation in people with cancer who have no therapeutic or prophylactic indication for anticoagulation. The Cochrane database of systematic reviews. 2017;12:CD006466.

Kahale L, Matar C, Tsolakian I, Hakoum M, Barba M, Yosuico V, et al. Oral anticoagulation in people with cancer who have no therapeutic or prophylactic indication for anticoagulation. Cochrane Database of Systematic Reviews [Internet]. 2021; Available from: <http://dx.doi.org/10.1002/14651858.CD006466.pub7>

1. Kaka 2022

Kaka AS, Duan-Porter W, Wilt TJ, MacDonald R, Greer N, Vela K, et al. Major Update: Remdesivir for Adults With COVID-19 : A Living Systematic Review and Meta-analysis for the American College of Physicians Practice Points. Annals of internal medicine [Internet]. 2021; Available from: http://ovidsp.ovid.com/ovidweb.cgi?T=JS&PAGE=reference&D=emexb&NEWS=N&AN=634244795

Wilt TJ, Kaka AS, MacDonald R, Greer N, Obley A, Duan-Porter W. Remdesivir for Adults With COVID-19 : A Living Systematic Review for American College of Physicians Practice Points. Annals of internal medicine. 2021;174:209–20.

Kaka A.S., MacDonald R., Linskens E.J., Langsetmo L., Vela K., Duan-Porter W., et al. Major Update 2: Remdesivir for Adults With COVID-19: A Living Systematic Review and Meta-analysis for the American College of Physicians Practice Points. Annals of Internal Medicine. 2022;175:701–9.

Anonymous. Erratum: Major update: remdesivir for adults with COVID-19. A living systematic review and meta-analysis for the American College of Physicians practice points (Ann Intern Med. (2021) 174 (663-672)) DOI: 10.7326/M20-8148). Annals of Internal Medicine. 2022;175:458.

1. Kallmes 2022

Kallmes K., Cowie K., Hardy N., Holub K. Software Tools For Systematic Literature Review In Medicine: A Review And Feature Analysis. International Journal of Technology Assessment in Health Care. 2022;38:S27.

1. Kashur 2021

Kashour T, Tleyjeh IM. Efficacy and safety of tocilizumab in COVID-19 patients: a living systematic review and meta-analysis - Author’s reply. Clinical microbiology and infection : the official publication of the European Society of Clinical Microbiology and Infectious Diseases [Internet]. 2021; Available from: <http://ovidsp.ovid.com/ovidweb.cgi?T=JS&PAGE=reference&D=medp&NEWS=N&AN=33705848>

1. Khalil 2022

Khalil H., Littmoden M., Gupta A., Banjoko A., Ansari K., Kumaran M.K., et al. Risk factors for SARS-CoV- 2 Positivity in neonates born to mothers with COVID-19. BJOG: An International Journal of Obstetrics and Gynaecology. 2022;129:141.

1. Khalili 2020

Khalili M, Chegeni M, Javadi S, Farokhnia M, Sharifi H, Karamouzian M. Therapeutic interventions for COVID-19: a living overview of reviews. Therapeutic advances in respiratory disease. 2020;14:1753466620976021.

1. Khamis 2019

Khamis AM, Kahale LA, Pardo-Hernandez H, Schunemann HJ, Akl EA. LSR_data abstraction form_20190129.xlsx. Figshare. 2019;

1. Khan 2023

Khan M, Bin Riaz I, Naqvi S, Saeed S, Faridi S, Khakwani K, et al. Toxicity Profile of Janus Kinase Inhibitors (JAKi) in Patients with Myelofibrosis: A Report from a Living Systematic Review and Meta-Analysis. BLOOD. 2023;142.

1. Khashaba 2023

Khashaba A, Barry K, Mamey M, Yap M, Sheikh J, Allotey J. Risk factors associated with maternal death in pregnant women with SARS-CoV-2 infection: Living systematic review and meta-analysis. BJOG-AN INTERNATIONAL JOURNAL OF OBSTETRICS AND GYNAECOLOGY. 2023;130:24–5.

1. Kim 2022

Kim J., Sung H., Lee H., Kim J.-S., Shin S., Jeong S., et al. Clinical Performance of Rapid and Point-of-Care Antigen Tests for SARS-CoV-2 Variants of Concern: A Living Systematic Review and Meta-Analysis. Viruses. 2022;14:1479.

1. Kimber 2021

Kimber C, Valk S, Chai K, Piechotta V, Iannizzi C, Monsef I, et al. Hyperimmune immunoglobulin for people with COVID‐19. Cochrane Database of Systematic Reviews [Internet]. 2021; Available from: <http://dx.doi.org/10.1002/14651858.CD015167>

Kimber C, Valk S, Chai K, Piechotta V, Iannizzi C, Monsef I, et al. Hyperimmune immunoglobulin for people with COVID‐19. Cochrane Database of Systematic Reviews [Internet]. 2023; Available from: http://dx.doi.org/10.1002/14651858.CD015167.pub2

1. Kirkovski 2021

Kirkovski A, Meyerowitz-Katz G. Ivermectin for treatment and prevention of COVID-19: A living systematic review. Open Science Framework. 2021;

1. Knight 2023

Knight C, Akande O, Khalil H, Kumaran M, Sheikh J, Allotey J, et al. Timing of mother-to-child transmission of SARS-CoV-2: living systematic review and meta-analysis (update). BJOG-AN INTERNATIONAL JOURNAL OF OBSTETRICS AND GYNAECOLOGY. 2023;130:167–8.

1. Knipe 2022

Knipe D, John A, Padmanathan P, Eyles E, Dekel D, Higgins JPT, et al. Suicide and self-harm in low- and middle- income countries during the COVID-19 pandemic: A systematic review. PLOS global public health. 2022;2:e0000282.

1. Koeckerling 2021

Koeckerling D, Pan D, Barker J. Re: “Efficacy and safety of tocilizumab in COVID-19 patients: a living systematic review and meta-analysis” by Tleyjeh et al. Clinical microbiology and infection : the official publication of the European Society of Clinical Microbiology and Infectious Diseases [Internet]. 2021; Available from: <http://ovidsp.ovid.com/ovidweb.cgi?T=JS&PAGE=reference&D=medp&NEWS=N&AN=33549765>

1. Kolakowsky-Hayner 2017

Kolakowsky-Hayner SA, Balamane M, Totten AM, Assasnik S, Ghajar J. Living guideline development in traumatic brain injury: Opportunities to link quality of care, research, and better patient outcomes to a future research agenda. Journal of Head Trauma Rehabilitation. 2017;32:E85–6.

1. Konnyu 2023

Konnyu K, Yogasingam S, Lépine J, Sullivan K, Alabousi M, Edwards A, et al. Quality improvement strategies for diabetes care: Effects on outcomes for adults living with diabetes. Cochrane Database of Systematic Reviews [Internet]. 2023; Available from: http://dx.doi.org/10.1002/14651858.CD014513

1. Korang 2022

Korang SK, Von Rohden E, Veroniki AA, Ong G, Ngalamika O, Siddiqui F, et al. Randomized clinical trials included in systematic review of vaccines against COVID-19. Figshare. 2022.

1. Kramer 2022

Kramer A, Prinz C, Fichtner F, Fischer A-L, Thieme V, Grundeis F, et al. Janus kinase inhibitors for the treatment of COVID‐19. Cochrane Database of Systematic Reviews [Internet]. 2022; Available from: <http://dx.doi.org/10.1002/14651858.CD015209>

1. Kreuzberger 2021a

Kreuzberger N, Hirsch C, Chai KL, Piechotta V, Valk SJ, Estcourt LJ, et al. SARS‐CoV‐2‐neutralising monoclonal antibodies for treatment of COVID‐19. Cochrane Database of Systematic Reviews [Internet]. 2021; Available from: <http://dx.doi.org/10.1002/14651858.CD013825>

1. Kreuzberger 2021b

Kreuzberger N, Hirsch C, Vanshylla K, Di Cristanziano V, Dorando E, Khosravi Z, et al. Persistence of immunoglobulin G after natural infection with SARS‐CoV‐2. Cochrane Database of Systematic Reviews [Internet]. 2021; Available from: <http://dx.doi.org/10.1002/14651858.CD014946>

1. Kuhne 2022

Kuhne L., Brussermann S., De Santis K.K., Jackle S., Grimm S., Ha T.-H., et al. EsteR - Decision support for German health departments by risk modelling in order to contain the COVID-19 pandemic. A rapid living review. Gesundheitswesen, Supplement. 2022;84:812–3.

1. Kulper-Schiek 2022

Kulper-Schiek W., Piechotta V., Pilic A., Batke M., Dreveton L.-S., Geurts B., et al. Facing the Omicron variant - How well do vaccines protect against mild and severe COVID-19? Third interim analysis of a living systematic review. medRxiv [Internet]. 2022; Available from: <https://www.medrxiv.org/>

1. Kumaran 2022

Kumaran M., Akande O., Khalil H., Lawson H., Sheikh J., Allotey J., et al. Mother-to- child transmission of SARS-CoV- 2 and rates of neonatal positivity. BJOG: An International Journal of Obstetrics and Gynaecology. 2022;129:149.

1. Laine 2023

Laine C, Chang S. Getting to the Truth About the Effectiveness of Masks in Preventing COVID-19. ANNALS OF INTERNAL MEDICINE. 2023;176:870–1.

1. Lam 2023

Lam M, Spuls P, Leshem Y, Gerbens L, Thomas K, Arents B, et al. Reporting of Harmonising Outcome Measures for Eczema (HOME) core outcome set instruments in randomized clinical trials for systemic treatments in atopic dermatitis. BRITISH JOURNAL OF DERMATOLOGY. 2023;189:494–6.

1. Lane 2022

Lane S., Yeomans A., Shakir S. Myocarditis and pericarditis in immunocompromised individuals following COVID-19 mRNA vaccination: A living review of spontaneous reports. Pharmacoepidemiology and Drug Safety. 2022;31:622–3.

1. Lang 2021

Lang A, Schlesinger S, Neuenschwander M, Pafili K, Kuss O, Herder C, et al. Risk phenotypes of diabetes and association with COVID-19 severity and death: a living systematic review and meta-analysis. Diabetologia [Internet]. 2021; Available from: <http://link.springer.de/link/service/journals/00125/index.htm>

Schlesinger S, Lang A, Christodoulou N, Linnerz P, Pafili K, Kuss O, et al. Risk phenotypes of diabetes and association with COVID-19 severity and death: an update of a living systematic review and meta-analysis. DIABETOLOGIA. 2023;66:1395–412.

1. Langford 2020

Langford BJ, So M, Raybardhan S, Leung V, Westwood D, MacFadden DR, et al. Bacterial co-infection and secondary infection in patients with COVID-19: a living rapid review and meta-analysis. Clinical Microbiology and Infection. 2020;26:1622‐1629.

1. Langford 2022

Langford B.J., So M., Leung V., Raybardhan S., Lo J., Kan T., et al. Predictors and microbiology of respiratory and bloodstream bacterial infection in patients with COVID-19: living rapid review update and meta-regression. Clinical Microbiology and Infection. 2022;28:491–501.

1. LaRosa 2023

La Rosa G, Vernooij R, Qureshi M, Polosa R, O’Leary R. Clinical testing of the cardiovascular effects of e-cigarette substitution for smoking: a living systematic review. INTERNAL AND EMERGENCY MEDICINE. 2023;18:917–28.

1. Lawrenson 2021

Lawrenson JG, Dhakal R, Verkicharla PK, Shah R, Huntjens B, Downie LE, et al. Interventions for myopia control in children: a living systematic review and network meta‐analysis. Cochrane Database of Systematic Reviews [Internet]. 2021; Available from: <http://dx.doi.org/10.1002/14651858.CD014758>

Lawrenson J, Shah R, Huntjens B, Downie L, Virgili G, Dhakal R, et al. Interventions for myopia control in children: a living systematic review and network meta‐analysis. Cochrane Database of Systematic Reviews [Internet]. 2023; Available from: http://dx.doi.org/10.1002/14651858.CD014758.pub2

1. Lawson 2021

Lawson H, Sheikh J, Yap M, Chatterjee S, Kew T, Debenham L, et al. Maternal and perinatal outcomes in pregnant and recently pregnant women with coronavirus disease 2019: living systematic review and meta-analysis. Bjog-an International Journal of Obstetrics and Gynaecology. 2021;128:81–2.

1. Lee 2021a

Lee V.Y., Monjur M.R., Gupta Y., Goyal A., Di Tanna G.L., Tandon N., et al. Interventions to prevent or delay the onset of type 2 diabetes in women with prior gestational diabetes mellitus: Protocol for a living systematic review and prospective meta-analysis. medRxiv [Internet]. 2021; Available from: <https://www.medrxiv.org/>

1. Lee 2021b

Lee Y., Kirubarajan A., Patro N., Soon M.S., Doumouras A.G., Hong D. Impact of hospital lockdown secondary to COVID-19 and past pandemics on surgical practice: A living rapid systematic review. American Journal of Surgery. 2021;222:67–85.

1. Legare 2021

Legare F., Plourde K.V., Charif A.B., Gogovor A., Brundisini F.K., McLean R.K.D., et al. Evidence on scaling in health and social care: protocol for a living umbrella review. Systematic Reviews. 2021;10:261.

1. Lewis 2016

Lewis SZ, Newton M. Innovative approach to keeping evidence current and guidelines living. Value in Health. 2016;19:A397.

1. Li 2023

Li C, Stebbins R, Noppert G, Carney C, Liu C, Sapp A, et al. Peripheral immune function and Alzheimer’s disease: a living systematic review and critical appraisal. MOLECULAR PSYCHIATRY. 2023;

1. Lindson 2023

Lindson N, Butler A, Liber A, Levy D, Barnett P, Theodoulou A, et al. An exploration of flavours in studies of e-cigarettes for smoking cessation: secondary analyses of a systematic review with meta-analyses. ADDICTION. 2023;118:634–45.

1. Lindson 2024

Lindson N, Butler A, McRobbie H, Bullen C, Hajek P, Begh R, et al. Electronic cigarettes for smoking cessation. Cochrane Database of Systematic Reviews [Internet]. 2024; Available from: http://dx.doi.org/10.1002/14651858.CD010216.pub8

1. Lithopoulos 2022

Lithopoulos A, Bayley M, Curran D, Fischer L, Knee C, Lauzon J, et al. Protocol for a living systematic review for the management of concussion in adults. BMJ open. 2022;12:e061282.

1. Littlewood 2022

Littlewood E, McMillan D, Chew Graham C, Bailey D, Gascoyne S, Sloane C, et al. Can we mitigate the psychological impacts of social isolation using behavioural activation? Long-term results of the UK BASIL urgent public health COVID-19 pilot randomised controlled trial and living systematic review. Evidence-based mental health. 2022;25:e49–57.

1. Liu 2022

Liu R., Patel A., Du X., Liu H., Liu B., Wang C., et al. Association between influenza vaccination, all-cause mortality and cardiovascular mortality: a protocol for a living systematic review and prospective meta-analysis. BMJ Open. 2022;12:e054171.

Liu R, Fan Y, Patel A, Liu H, Du X, Liu B, et al. The association between influenza vaccination, cardiovascular mortality and hospitalization: A living systematic review and prospective meta-analysis. VACCINE. 2024;42:1034–41. LobbÃ© 2022

1. Lu 2021

Lu C, Lu T, Pan B, Wang Q, Hou L, Zhang Q, et al. Coronavirus disease (COVID 2019): protocol for a living overview of systematic reviews. Annals of palliative medicine. 2021;10:1488–93.

1. Lujintanon 2023

Lujintanon S. Commonality and co-occurrence of discrete strategies within implementation strategy bundles: results from a living review of global hiv implementation research. IMPLEMENTATION SCIENCE. 2023;18.

1. Mackey 2020

Mackey K, King VJ, Gurley S, Kiefer M, Liederbauer E, Vela K, et al. Risks and Impact of Angiotensin-Converting Enzyme Inhibitors or Angiotensin-Receptor Blockers on SARS-CoV-2 Infection in Adults: A Living Systematic Review. Annals of internal medicine. 2020;173:195–203.

1. Mackey 2021

Mackey K, Arkhipova-Jenkins I, Armstrong C, Gean E, Anderson J, Paynter RA, et al. Antibody Response Following SARS-CoV-2 Infection and Implications for Immunity: A Rapid Living Review. 2021;

1. Macura 2021

Macura B, Thomas J, Metson GS, McConville JR, Johannesdottir SL, Seddon D, et al. Additional file 3 of Technologies for recovery and reuse of plant nutrients from human excreta and domestic wastewater: a protocol for a systematic map and living evidence platform. Figshare. 2021.

Macura B, Thomas J, Metson GS, McConville JR, Johannesdottir SL, Seddon D, et al. Additional file 4 of Technologies for recovery and reuse of plant nutrients from human excreta and domestic wastewater: a protocol for a systematic map and living evidence platform. Figshare. 2021.

1. Maguire 2020

Maguire BJ. A Living Systematic Review for COVID-19 Clinical Trial Registrations. Open Science Framework. 2020;

Maguire B. Associated data for: IDDO Living Systematic Review for COVID-19 Clinical Trial Registrations. Harvard Dataverse. 2020;

1. Malin 2022

Malin J.J., Spinner C.D., Janssens U., Welte T., Weber-Carstens S., Schalte G., et al. Correction to: Key summary of German national treatment guidance for hospitalized COVID-19 patients: Key pharmacologic recommendations from a national German living guideline using an Evidence to Decision Framework (last updated 17.05.2021) (Infection, (2. Infection. 2022;50:107–8.

Malin J.J., Spinner C.D., Janssens U., Welte T., Weber-Carstens S., Schalte G., et al. Key summary of German national treatment guidance for hospitalized COVID-19 patients: Key pharmacologic recommendations from a national German living guideline using an Evidence to Decision Framework (last updated 17.05.2021). Infection. 2022;50:93–106.

1. Margherita 2023

Margherita G, Caffieri A. An observatory on changes in dreaming during a pandemic: a living systematic review (part 1). JOURNAL OF SLEEP RESEARCH. 2023;32.

1. Matar 2021

Matar C. MM-450: Anti-Thrombotic Therapy for Ambulatory Patients with Multiple Myeloma Receiving Immunomodulatory Agents: A Systematic Review. Clinical Lymphoma, Myeloma and Leukemia. 2021;21:S445.

1. Mathioudakis 2022

Mathioudakis A. Update to living systematic review on effectiveness of heterologous and homologous covid-19 vaccine regimens. BMJ-BRITISH MEDICAL JOURNAL. 2022;379.

1. McDonagh 2020

McDonagh MS, Wagner J, Ahmed AY, Morasco B, Kansagara D, Chou R. Living Systematic Review on Cannabis and Other Plant-Based Treatments for Chronic Pain - Quarterly Progress Report: December 2020. 2020; Available from: <http://ovidsp.ovid.com/ovidweb.cgi?T=JS&PAGE=reference&D=medp&NEWS=N&AN=33764726>

Chou R, Wagner J, Ahmed AY, Morasco BJ, Kansagara D, Selph S, et al. Living Systematic Review on Cannabis and Other Plant-Based Treatments for Chronic Pain: 2022 Update. 2022;

1. McFadyen 2021

McFadyen CA, Zeiler FA, Newcombe V, Synnot A, Steyerberg E, Gruen RL, et al. Apolipoprotein E4 Polymorphism and Outcomes from Traumatic Brain Injury: A Living Systematic Review and Meta-Analysis. Journal of neurotrauma. 2021;38:1124–36.

1. Melo 2021

Melo A.K.G., Milby K.M., Caparroz A.L.M.A., Pinto A.C.P.N., Santos R.R.P., Rocha A.P., et al. Biomarkers of cytokine storm as red flags for severe and fatal COVID-19 cases: A living systematic review and meta-analysis. PLoS ONE. 2021;16:e0253894.

1. Michelen 2022

Michelen M., Sigfrid L., Kartsonaki C., Shemilt I., Hastie C., O’Hara M.E., et al. Characterising Long Covid: a living systematic review update with controlled studies. medRxiv [Internet]. 2022; Available from: https://www.medrxiv.org/

1. Mikolajewska 2021

Mikolajewska A, Fischer A-L, Piechotta V, Mueller A, Metzendorf M-I, Becker M, et al. Colchicine for the treatment of COVID‐19. Cochrane Database of Systematic Reviews [Internet]. 2021; Available from: <http://dx.doi.org/10.1002/14651858.CD015045>

1. Miller 2023

Miller S, Keenan C, Early E, McConnell K, Rodriguez L. PROTOCOL: Education and Covid-19: An evidence and gap map. CAMPBELL SYSTEMATIC REVIEWS. 2023;19.

1. Milovanovic 2021

Milovanovic L, Bagshaw SM, Rewa O, Hessey E, Sebastianski M, Keto-Lambert Di, et al. Epidemiology, clinical characteristics and treatment of critically ill patients with COVID-19): A protocol for a living systematic review. BMJ open. 2021;11:042008.

1. MisinformationLivingEvidence 2023

Misinformation Living Evidence, Wilson M, Vélez M, Lavis J. Impact of strategies to mitigate misinformation in diverse settings and populations: a protocol for a living evidence synthesis. BMJ OPEN. 2023;13.

1. Mohler 2023

Möhler R, Calo S, Renom A, Renom H, Meyer G. Personally tailored activities for improving psychosocial outcomes for people with dementia in long‐term care. Cochrane Database of Systematic Reviews [Internet]. 2023; Available from: http://dx.doi.org/10.1002/14651858.CD009812.pub3

1. Mol 2022

Mol CG, Vieira AG da S, Garcia BMSP, Pereira EDS, Eid RAC, Pinto ACPN, et al. Closed-loop oxygen control for patients with hypoxaemia during hospitalisation: a living systematic review and meta-analysis protocol. BMJ open. 2022;12:e062299.

1. Moneer 2022

Moneer O., Daly G., Skydel J.J., Nyhan K., Lurie P., Ross J.S., et al. Agreement of treatment effects from observational studies and randomized controlled trials evaluating hydroxychloroquine, lopinavir-ritonavir, or dexamethasone for covid-19: meta-epidemiological study. BMJ (Clinical research ed). 2022;377:e069400.

1. Moore 2020

Moore AJ, Nakahata MI, Kalinich CC, Nyhan K, Bromberg DJ, Shi X, et al. The Sensitivity of Respiratory Tract Specimens for the Detection of SARS-CoV-2: A Protocol for a Living Systematic Review and Meta-Analysis. medRxiv : the preprint server for health sciences [Internet]. 2020; Available from: <http://ovidsp.ovid.com/ovidweb.cgi?T=JS&PAGE=reference&D=pmnm&NEWS=N&AN=32637978>

1. Morales 2021

Morales MB, Ortiz-Munoz L, Duarte Anselmi G, Rada G, Covid- L. Ove Working Group. Use of gloves for the prevention of COVID-19 in healthy population: A living systematic review protocol. Health science reports. 2021;4:e255.

Ortiz-Munoz LE, Ferrer MBM, Anselmi GD, Rada G. Protocol of a Living systematic review: Gloves for the prevention of COVID-19 in healthy population. Open Science Framework. 2020;

1. Morey 2022

Morey J., Erdman J., Zhang X., Kim B., Redlener M., Fifi J. 26 Emergent Large Vessel Occlusion Stroke Direct Triage Models: A Systematic Review and Meta-Analysis. Annals of Emergency Medicine. 2022;80:S12.

1. Morsi 2023

Morsi R, Elfil M, Ghaith H, Aladawi M, Elmashad A, Kothari S, et al. Endovascular Thrombectomy for Large Ischemic Strokes: A Living Systematic Review and Meta-Analysis of Randomized Trials. JOURNAL OF STROKE. 2023;25:214-+.

1. Naqvi 2023ª

Naqvi S, Bin Riaz I, He H, Imran M, Orme J, Childs D, et al. The role of volume of disease for treatment selection in patients with metastatic castration sensitive prostate cancer (mCSPC): A living meta-analysis. JOURNAL OF CLINICAL ONCOLOGY. 2023;41.

1. Naqvi 2023b

Naqvi S, He H, Siddiqi R, Khan N, Khakwani K, Ayaz A, et al. Real-time evidence synthesis for first line (1L) treatment of metastatic renal cell carcinoma (mRCC): A living, interactive systematic review and Bayesian network metaanalysis. JOURNAL OF CLINICAL ONCOLOGY. 2023;41.

1. Nassar 2023

Nassar E, Abdulkareem D, Thombs B. Results from a living systematic review of the prevalence of mood and anxiety disorders and factors associated with symptoms in systemic sclerosis. SCIENTIFIC REPORTS. 2023;13.

1. Nassr 2022

Nassr N. Duration of Antibiotic Therapy for the Management of Urinary Tract Infectionsin Newborns and Young Infants Aged Under 3 Months: A Living Systematic Review and Meta-analysis Using Crowd sourcing. Pediatrics [Internet]. 2022;149. Available from: https://publications.aap.org/pediatrics/article/149/1MeetingAbstractsFebruary2022/540/186356/Duration-of-Antibiotic-Therapy-for-the-Management

1. Naughton 2022

Naughton S., Peeters A. Mapping the outcomes of the Centre of Research Excellence in food retail environments for health (RE-FRESH). Obesity Reviews. 2022;23.

1. NCT 04960241

NCT04960241. Does Rehabilitation After Total Hip Or Knee Arthroplasty Work (DRAW2). 2021; Available from: <https://www.cochranelibrary.com/central/doi/10.1002/central/CN-02290342/full>

1. Nedelcu 2023

Nedelcu A, Oerther B, Engel H, Sigle A, Schmucker C, Schoots I, et al. A Machine Learning Framework Reduces the Manual Workload for Systematic Reviews of the Diagnostic Performance of Prostate Magnetic Resonance Imaging. EUROPEAN UROLOGY OPEN SCIENCE. 2023;56:11–4.

1. Negrini 2022

Negrini S, Kiekens C, Cordani C, Arienti C, DE Groote W. Cochrane “evidence relevant to” rehabilitation of people with post COVID-19 condition. What it is and how it has been mapped to inform the development of the World Health Organization recommendations. European journal of physical and rehabilitation medicine. 2022;58:853–6.

1. Neves 2022

Neves H., Parente P., Gomes J., Queiros C., Sousa J., Parola V., et al. Nursing knowledge of people with paresis of voluntary muscles: a living scoping review protocol. JBI evidence synthesis. 2022;

1. Norton 2020

Norton A, Bucher A, Antonio E, Advani N, Grund H, Mburu S, et al. Replication data for “A living mapping review for COVID-19 funded research projects: three- month update.” Harvard Dataverse. 2020;1.

Norton A, Bucher A, Antonio E, Advani N, Grund H, Mburu S, et al. Replication Data for Baseline results of a living systematic review for COVID-19 funded research projects. Harvard Dataverse. 2020;1.

Norton A, Bucher A, Antonio E, Advani N, Grund H, Mburu S, et al. Extended data for ’A living mapping review for COVID-19 funded research projects: nine-month update. Figshare. 2021.

Norton A, Bucher A, Antonio E, Advani N, Grund H, Mburu S, et al. Repilcation data for “A living mapping review for COVID-19 funded research projects: nine-month update.” Harvard Dataverse. 2021.

Norton A, Bucher A, Antonio E, Grund H, Mburu S, Clegg E, et al. Replication Data for “A living mapping review for COVID-19 funded research projects: six-month update.” Harvard Dataverse. 2021.

Norton A, Bucher A, Antonio E, Advani N, Johnston C, Grund H, et al. Extended data for A living mapping review for COVID-19 funded research projects: one year update. Figshare. 2022.

Norton A, Bucher A, Antonio E, Advani N, Grund H, Mburu S, et al. Replication Data for A living mapping review for COVID-19 funded research projects: one year update. Harvard Dataverse. 2022;1.

1. Nussbaumer-Streit 2020

Nussbaumer-Streit B, Klerings I, Gartlehner G. Reply to letter to the editor “Lessons from COVID-19 to future evidence synthesis efforts: first living search strategy and out of date scientific publishing and indexing industry.” Journal of clinical epidemiology. 2020;123:173–4.

1. Nyirenda 2022

Nyirenda J.L.Z., Sofroniou M., Toews I., Mikolajewska A., Lehane C., Monsef I., et al. Fluvoxamine for the treatment of COVID-19. Cochrane Database of Systematic Reviews. 2022;2022:CD015391.

1. Oikonomidi 2020

Oikonomidi T, Boutron I, Pierre O, Cabanac G, Ravaud P. Changes in evidence for studies assessing interventions for COVID-19 reported in preprints: meta-research study. BMC medicine. 2020;18:402.

1. O'Byrne 2022

O’Byrne L, Webster KE, MacKeith S, Philpott C, Hopkins C, Burton MJ. Interventions for the treatment of persistent post-COVID-19 olfactory dysfunction. The Cochrane database of systematic reviews. 2022;9:CD013876.

1. O'Leary 2023

O’Leary R, La Rosa G, Vernooij R, Polosa R. Identifying spin bias of nonsignificant findings in biomedical studies. BMC RESEARCH NOTES. 2023;16.

1. Oerther 2022

Oerther B, Schmucker C, Schwarzer G, Schoots I, Sigle A, Gratzke C, et al. Living systematic review and meta-analysis of the prostate MRI diagnostic test with Prostate Imaging Reporting and Data System (PI-RADS) assessment for the detection of prostate cancer: study protocol. BMJ open. 2022;12:e066327.

1. Ortiz-Munoz 2020

Ortiz-Munoz LE, Verdugo-Paiva F, Bravo-Jeria R, Morel M, Acuna MP, Rada G. Ivermectin for COVID-19: A living systematic review project. Open Science Framework. 2020;

1. Ozlusen 2021

Ozlusen B., Kozan S., Akcan R.E., Kalender M., Yaprak D., Peltek I.B., et al. Effectiveness of favipiravir in COVID-19: a live systematic review. European Journal of Clinical Microbiology and Infectious Diseases. 2021;40:2575–83.

1. Panara 2021

Panara K, Kumari R. Data set for AYUSH interventions for COVID-19- A Living Systematic Review and Meta-analysis. Zenodo. 2021.

Panara K, Kumari R. Prisma P Checklist of protocol - AYUSH interventions for COVID-19 - A Living Systematic Review and Meta-analysis. Zenodo. 2021.

1. Parker 2019

Parker N, Al-Obaidi A, Van Truong Q, Badgett R. Pembrolizumab versus the standard of care for cancer therapy: A meta-analysis of 12 KEYNOTE trials comparing overall survival. Journal of Clinical Oncology [Internet]. 2019;37. Available from: <https://ascopubs.org/doi/abs/10.1200/JCO.2019.37.15_suppl.e14159>

1. Patnode 2021

Patnode C, Henrikson NB, Kansagara D, Chou R, Laine C. Rapid and Living Reviews in the Time of Covid-19. Annals of Behavioral Medicine. 2021;55:S611–S611.

1. Paul 2021

Paul A., Abulannaz O., Chhabra M., Le M.L., Kelly L.E. PIH19 Dosing and Safety of Medical Cannabis in Children: Preliminary Findings from a Living Systematic Review. Value in Health. 2021;24:S102.

1. Pearson 2021

Pearson CF, Jeffery R, Oxford-Cardiff C-LC, Thornton EE. Mucosal immune responses in COVID19 - a living review. Oxford open immunology. 2021;2:iqab002.

1. Pelletier 2005

Pelletier KR. A Review and Analysis of the Clinical and Cost-Effectiveness Studies of Comprehensive Health Promotion and Disease Management Programs at the Worksite: Update VI 2000???2004. Journal of Occupational and Environmental Medicine. 2005;47:1051–8.

1. Penserga 2021

Penserga E. The living recommendations paradigm in OA treatment guidelines: Navigating the dissonance. International Journal of Rheumatic Diseases. 2021;24:22.

1. Pham 2022

Pham B., Rios P., Radhakrishnan A., Darvesh N., Antony J., Williams C., et al. Comparative-effectiveness research of COVID-19 treatment: a rapid scoping review. BMJ Open. 2022;12:e045115.

1. Picot 2023

Picot C, Ajiji P, Jurek L, Nourredine M, Massardier J, Peron A, et al. Risk of drug use during pregnancy: master protocol for living systematic reviews and meta-analyses performed in the metaPreg project. SYSTEMATIC REVIEWS. 2023;12.

1. Pillay 2022

Pillay J, Gaudet L, Wingert A, Bialy L, Mackie AS, Paterson DI, et al. Incidence, risk factors, natural history, and hypothesised mechanisms of myocarditis and pericarditis following covid-19 vaccination: living evidence syntheses and review. BMJ (Clinical research ed). 2022;378:e069445.

1. Popp 2021

Popp M, Stegemann M, Riemer M, Metzendorf M-I, Romero C, Mikolajewska A, et al. Antibiotics for the treatment of COVID‐19. Cochrane Database of Systematic Reviews [Internet]. 2021; Available from: <http://dx.doi.org/10.1002/14651858.CD015025>

1. Popp 2022

Popp M, Reis S, Schießer S, Hausinger Ri, Stegemann M, Metzendorf M-I, et al. Ivermectin for preventing and treating COVID‐19. Cochrane Database of Systematic Reviews [Internet]. 2022; Available from: <http://dx.doi.org/10.1002/14651858.CD015017.pub3>

1. PopulationHlthMedSciCommAmer 2023

Population Hlth Med Sci Comm Amer, Qaseem A, Yost J, Abraham G, Andrews R, Jokela J, et al. Outpatient Treatment of Confirmed COVID-19: Living, Rapid Practice Points From the American College of Physicians (Version 2). ANNALS OF INTERNAL MEDICINE. 2023;176:1396-+.

1. Pourali 2020

Pourali F, Afshari M, Alizadeh-Navaei R, Javidnia J, Moosazadeh M, Hessami A. Relationship between blood group and risk of infection and death in COVID-19: a live meta-analysis. New microbes and new infections. 2020;37:100743.

1. Probst 2019

Probst P, Hüttner FJ, Meydan Ö, Kalkum E, Kretschmer R, Jensen K, et al. Evidence map of pancreatic surgery: protocol for a living systematic review and meta-analysis. BMJ Open. 2019;9:e032353.

Probst P, Hüttner FJ, Meydan Ö, Abu Hilal M, Adham M, Barreto SG, et al. Evidence Map of Pancreatic Surgery–A living systematic review with meta-analyses by the International Study Group of Pancreatic Surgery (ISGPS). Surgery. 2021;170:1517–24.

1. Qaseem 2020

Qaseem A, Yost J, Etxeandia-Ikobaltzeta I, Miller MC, Abraham GM, Obley AJ, et al. Should Clinicians Use Chloroquine or Hydroxychloroquine Alone or in Combination With Azithromycin for the Prophylaxis or Treatment of COVID-19? Living Practice Points From the American College of Physicians (Version 1). Annals of internal medicine. 2020;173:137-

1. Qiu 2021

Qiu X, Nergiz AI, Maraolo AE, Bogoch II, Low N, Cevik M. The role of asymptomatic and pre-symptomatic infection in SARS-CoV-2 transmission-a living systematic review. Clinical microbiology and infection : the official publication of the European Society of Clinical Microbiology and Infectious Diseases. 2021;27:511–9.

1. Quispe 2020

Quispe GJPB, Vasquez AAH, Comande D. Retracted publications about COVID-19 in scientific journals: A living systematic review. Open Science Framework. 2020;

1. Ramanadhan 2023

Ramanadhan S, Hansen K, Henderson J, Cohen M, Paynter R, Edelman A. Risk of thromboembolism in patients with COVID‐19 who are using hormonal contraception. Cochrane Database of Systematic Reviews [Internet]. 2023; Available from: http://dx.doi.org/10.1002/14651858.CD014908.pub3

1. Rayner 2023a

Rayner D, Nunes J, Chu A, Orchanian-Cheff A, Foroutan F, Rotstein C, et al. COVID-19 Vaccination Strategies in Solid Organ Transplant Recipients: A Living Systematic Review and Network Meta-Analysis. JOURNAL OF HEART AND LUNG TRANSPLANTATION. 2023;42:S13–S13.

1. Rayner 2023b

Rayner D, Nunes J, Chu A, Jamil Y, Orchanian-Cheff A, Foroutan F, et al. COVID-19 Vaccination Strategies and Seroconversion in Solid Organ Transplant Recipients: A Living Systematic Review and Network Meta-Analysis of Observational Studies. AMERICAN JOURNAL OF TRANSPLANTATION. 2023;23:S668–S668.

1. Rayner 2023c

Rayner D, Nunes J, Chu A, Jamil Y, Orchanian-Cheff A, Rotstein C, et al. Number of COVID-19 Vaccine Doses and Clinical Outcomes in Solid Organ Transplant Recipients: A Living Systematic Review and Network Meta-Analysis of Observational Studies. AMERICAN JOURNAL OF TRANSPLANTATION. 2023;23:S669–S669.

1. Renton 2022

Renton W, Tiller G, Munro J, Tan J, Johnston R, Avery J, et al. Dose reduction and discontinuation of disease‐modifying anti‐rheumatic drugs (DMARDs) for juvenile idiopathic arthritis. Cochrane Database of Systematic Reviews [Internet]. 2022; Available from: <http://dx.doi.org/10.1002/14651858.CD014961>

1. Reed 2022

Reed AC, Murmann M, Ramzy A, Scott M, Skidmore B, Welch V, et al. PROTOCOL: Exploring education to support vaccine confidence amongst healthcare and long-term care staff amidst the COVID-19 pandemic: A protocol for a living scoping review. Campbell systematic reviews. 2022;18:e1293.

1. Reis 2023

Reis S, Metzendorf M-I, Kuehn R, Popp M, Gagyor I, Kranke P, et al. Nirmatrelvir combined with ritonavir for preventing and treating COVID‐19. Cochrane Database of Systematic Reviews [Internet]. 2023; Available from: http://dx.doi.org/10.1002/14651858.CD015395.pub3

1. Reynolds 2020

Reynolds PS. When Evidence Goes “Missing in Action”: Implications for Patient Management in Cardiac Surgery. The journal of extra-corporeal technology. 2020;52:126–34.

1. Riaz 2022a

Riaz I.B., Sipra Q.U.A.R., Naqvi S.A.A., He H., Siddiqi R., Islam M., et al. Quantifying absolute benefit for adjuvant treatment options in renal cell carcinoma: A living interactive systematic review and network meta-analysis. Critical Reviews in Oncology/Hematology. 2022;175:103706.

1. Riaz 2022b

Riaz I.B., Naqvi S.A.A., He H., Asghar N., Siddiqi R., Khakwani K.Z.R., et al. 1417P Mixed treatment comparisons evaluating contemporary therapies in metastatic castration sensitive prostate cancer (mCSPC): A living systematic review. Annals of Oncology. 2022;33:S1193.

1. Riaz 2023

Riaz I, Naqvi S, He H, Asghar N, Siddiqi R, Liu H, et al. First-line Systemic Treatment Options for Metastatic Castration-Sensitive Prostate Cancer A Living Systematic Review and Network Meta-analysis. JAMA ONCOLOGY. 2023;9:635–45.

1. Richter 2021

Richter FC, Alrubayyi A, Teijeira Crespo A, Oxford-Cardiff COVID-19 Literature Consortium, Hulin-Curtis S. Impact of obesity and SARS-CoV-2 infection: implications for host defence - a living review. Oxford open immunology. 2021;2:iqab001.

1. Rios 2020

Rios P, Darvesh N, Antony J, Radhakrishnan A, Tricco AC, Lachance CC, et al. Global evidence of gender inequity in academic health research: A living scoping review protocol. JBI evidence synthesis. 2020;18:2181–93.

1. Rochwerg 2021

Rochwerg B., Kawano-Dourado L., Qadir N. How trustworthy guidelines can impact outcomes. Current opinion in critical care. 2021;27:544–50.

1. Saiz 2023

Saiz L, Leache L, Gutiérrez-Valencia M, Erviti J, Reyes M. Efficacy and safety of chimeric antigen receptor T-cell (CAR-T) therapy in hematologic malignancies: a living systematic review on comparative studies. THERAPEUTIC ADVANCES IN HEMATOLOGY. 2023;14.

1. Samaha 2024

Samaha J, Romei V. Alpha-Band Frequency and Temporal Windows in Perception: A Review and Living Meta-analysis of 27 Experiments (and Counting). JOURNAL OF COGNITIVE NEUROSCIENCE. 2024;36:640–54.

1. Sanuade 2023

Sanuade C, Wu N, Joyal-Desmarais K, Ribeiro P, Vieira A, Stojanovic J, et al. LONG-TERM EFFECTIVENESS OF THE PRIMARY SERIES OF COVID-19 VACCINES AGAINST OMICRON USING FINDINGS FROM A RAPID LIVING SYSTEMATIC EVIDENCE SYNTHESIS AND META-ANALYSIS. INTERNATIONAL JOURNAL OF BEHAVIORAL MEDICINE. 2023;30:S55–S55.

1. Saueressig 2022

Saueressig T, Braun T, Steglich N, Diemer F, Zebisch J, Herbst M, et al. Primary surgery versus primary rehabilitation for treating anterior cruciate ligament injuries: a living systematic review and meta-analysis. British journal of sports medicine. 2022;56:1241–51.

1. Sbidian 2021

Sbidian E., Chaimani A., Garcia-Doval I., Doney L., Dressler C., Hua C., et al. Systemic pharmacological treatments for chronic plaque psoriasis: a network meta-analysis. Cochrane Database of Systematic Reviews. 2021;2021:CD011535.

Sbidian E, Chaimani A, Garcia-Doval I, Doney L, Dressler C, Hua C, et al. Systemic pharmacological treatments for chronic plaque psoriasis: a network meta‐analysis. Cochrane Database of Systematic Reviews [Internet]. 2022; Available from: <http://dx.doi.org/10.1002/14651858.CD011535.pub5>

1. Schunemann 2020

Schunemann HJ, Khabsa J, Solo K, Khamis AM, Brignardello-Petersen R, El-Harakeh A, et al. Ventilation Techniques and Risk for Transmission of Coronavirus Disease, Including COVID-19: A Living Systematic Review of Multiple Streams of Evidence. Annals of internal medicine. 2020;173:204–16.

1. Scourfield 2021

Scourfield DO, Reed SG, Quastel M, Alderson J, Bart VMT, Teijeira Crespo A, et al. The role and uses of antibodies in COVID-19 infections: a living review. Oxford open immunology. 2021;2:iqab003.

1. Shaver 2023a

Shaver N, Bennett A, Beck A, Vyas N, Zitiktye G, Lam E, et al. Performance of different rapid antigen testing strategies for SARS-CoV-2: A living rapid review. EUROPEAN JOURNAL OF CLINICAL INVESTIGATION. 2023;

1. Shaver 2023b

Shaver N, Katz M, Asamoah G, Linkins L, Abdelkader W, Beck A, et al. Protocol for a living evidence synthesis on variants of concern and COVID-19 vaccine effectiveness. VACCINE. 2023;41:6411–8.

1. Sheikh 2022

Sheikh J, Lawson H, Allotey J, Yap M, Balaji R, Kew T, et al. Global variations in the burden of SARS-CoV-2 infection and its outcomes in pregnant women by geographical region and country’s income status: a meta-analysis. BMJ global health. 2022;7.

1. Shepperd 2016

Shepperd S, Iliffe S, Doll HA, Clarke MJ, Kalra L, Wilson AD, et al. Admission avoidance hospital at home. Cochrane Database of Systematic Reviews [Internet]. 2016; Available from: <http://dx.doi.org/10.1002/14651858.CD007491.pub2>

1. Shi 2020

Shi J, Gao Y, Zhao L, Li Y, Yan M, Niu MM, et al. Prevalence of delirium, depression, anxiety, and post-traumatic stress disorder among COVID-19 patients: protocol for a living systematic review. Systematic reviews. 2020;9:258.

1. Siemens 2022

Siemens W, Nothacker J, Stadelmaier J, Meerpohl JJ, Schmucker C. Three out of four published systematic reviews on COVID-19 treatments were not registered and one-third of those registered were published: a meta-research study. Journal of clinical epidemiology. 2022;152:36–46.

1. Siemens 2024

Siemens W, Bantle G, Mahler S, Nothacker J, Stadelmaier J, Bitzer E, et al. Clinical and methodological implications for research elements in systematic reviews on COVID-19 treatment were often unstructured and under-reported: a metaresearch study. JOURNAL OF CLINICAL EPIDEMIOLOGY. 2024;166.

1. Silveira 2022

Silveira F.M., Mello A.L.R., da Silva Fonseca L., Dos Santos Ferreira L., Kirschnick L.B., Martins M.D., et al. Morphological and tissue-based molecular characterization of oral lesions in patients with COVID-19: A living systematic review. Archives of oral biology. 2022;136:105374.

1. Simons 2020

Simons D, Shahab L, Brown J, Perski O. The association of smoking status with SARS-CoV-2 infection, hospitalisation and mortality from COVID-19: A living rapid evidence review and Bayesian meta-analyses (version 11) - Data. Figshare. 2020;

Simons D, Shahab L, Brown J, Perski O. The association of smoking status with SARS-CoV-2 infection, hospitalisation and mortality from COVID-19: A living rapid evidence review with Bayesian meta-analyses (version 7). Addiction (Abingdon, England) [Internet]. 2020; Available from: <http://ovidsp.ovid.com/ovidweb.cgi?T=JS&PAGE=reference&D=emexa&NEWS=N&AN=633092525>

1. Simpson 2016

Simpson SQ, Gaines M, Hussein Y, Badgett RG. Early goal-directed therapy for severe sepsis and septic shock: A living systematic review. Journal of critical care. 2016;36:43–8.

1. Singh 2022a

Singh N., Temin S., Baker S., Blanchard E., Brahmer J.R., Celano P., et al. Therapy for Stage IV Non-Small-Cell Lung Cancer with Driver Alterations: ASCO Living Guideline. Journal of Clinical Oncology. 2022;40:3310–22.

1. Singh 2022b

Singh N., Temin S., Baker S., Blanchard E., Brahmer J.R., Celano P., et al. Therapy for Stage IV Non-Small-Cell Lung Cancer Without Driver Alterations: ASCO Living Guideline. Journal of Clinical Oncology. 2022;40:3323–43.

1. Singh-Phulgenda 2022

Singh-Phulgenda S, Rashan S, Harriss E, Dahal P, Naylor C, Brack M, et al. Infectious diseases data observatory (IDDO) visceral leishmaniasis library of clinical therapeutic studies: A protocol for a living systematic review of clinical studies. Harvard Dataverse. 2022.

1. Sinyor 2022

Sinyor M., Zaheer R., Webb R.T., Knipe D., Eyles E., Higgins J.P.T., et al. SARS-CoV-2 Infection and the Risk of Suicidal and Self-Harm Thoughts and Behaviour: A Systematic Review. Canadian Journal of Psychiatry [Internet]. 2022; Available from: <https://journals.sagepub.com/home/cpa>

1. Sipra 2022

Sipra Q.U.A.R., Riaz I.B., Naqvi S.A.A., He H., Liu H., Bryce A.H., et al. Treatment of cancer associated thrombosis: A living interactive systematic review and bayesian network meta-analysis. Journal of Clinical Oncology [Internet]. 2022;40. Available from: https://ascopubs.org/doi/pdf/10.1200/JCO.2022.40.16_suppl.e24070

1. Sipra 2023

Sipra Q, Bin Riaz I, Naqvi S, He H, Siddiqi R, Islam M, et al. Adjuvant immunotherapy in renal cell carcinoma: A living systematic review and network meta-analysis (NMA). JOURNAL OF CLINICAL ONCOLOGY. 2023;41.

1. Smith 2022

Smith E.R., Oakley E., He S., Zavala R., Ferguson K., Miller L., et al. Protocol for a sequential, prospective metaanalysis to describe coronavirus disease 2019 (COVID-19) in the pregnancy and postpartum periods. PLoS ONE. 2022;17:e0270150.

1. Smits 2020

Smits LJM, Wynants L, Van Calster B, Steyerberg EW, Takada T, Collins GS, et al. Prediction models for diagnosis and prognosis of covid-19: Systematic review and critical appraisal. The BMJ. 2020;369:m1328.

Wynants. Update to living systematic review on prediction models for diagnosis and prognosis of covid-19. Bmj-British Medical Journal [Internet]. 2020;370. Available from: <https://www.bmj.com/content/bmj/370/bmj.m2810.full.pdf>

Anonymous. Update to living systematic review on prediction models for diagnosis and prognosis of covid-19. BMJ (Clinical research ed). 2021;372:n236.

Anonymous. Update to living systematic review on prediction models for diagnosis and prognosis of covid-19. BMJ (Clinical research ed). 2022;378:o2009.

1. Sommer 2023

Sommer I, Dobrescu A, Ledinger D, Moser I, Thaler K, Persad E, et al. Outpatient Treatment of Confirmed COVID-19: A Living, Rapid Review for the American College of Physicians. ANNALS OF INTERNAL MEDICINE. 2023;176:92-+.

Sommer I, Ledinger D, Thaler K, Dobrescu A, Persad E, Fangmeyer M, et al. Outpatient Treatment of Confirmed COVID-19: A Living, Rapid Evidence Review for the American College of Physicians (Version 2). ANNALS OF INTERNAL MEDICINE. 2023;176:1377-+.

1. Soto-Camara 2021

Soto-Camara R., Garcia-Santa-basilia N., Onrubia-Baticon H., Cardaba-Garcia R.M., Jimenez-Alegre J.J., Reques-Marugan A.M., et al. Psychological impact of the COVID-19 pandemic on out-of-hospital health professionals: A living systematic review. Journal of Clinical Medicine. 2021;10:5578.

1. Spurling 2023

Spurling G, Dooley L, Clark J, Askew D. Immediate versus delayed versus no antibiotics for respiratory infections. Cochrane Database of Systematic Reviews [Internet]. 2023; Available from: http://dx.doi.org/10.1002/14651858.CD004417.pub6

1. Stanford 2022

Stanford V, Gresh L, Toledo J, Mendez J, Aldighieri S, Reveiz L. Evidence in decision-making in the context of COVID-19 in Latin America. Lancet regional health Americas. 2022;14:100322.

1. Stroehlein 2021

Stroehlein JK, Wallqvist J, Iannizzi C, Mikolajewska A, Metzendorf MI, Benstoem C, et al. Vitamin D supplementation for the treatment of COVID‐19: a living systematic review. Cochrane Database of Systematic Reviews [Internet]. 2021; Available from: http://dx.doi.org/10.1002/14651858.CD015043

Stroehlein JK, Wallqvist J, Iannizzi C, Mikolajewska A, Metzendorf M-I, Benstoem C, et al. Risk of bias assessments for the Cochrane review “Vitamin D supplementation for the treatment of COVID-19: a living systematic review.” Zenodo. 2021.

1. Struyf 2022

Struyf T, Deeks J, Dinnes J, Takwoingi Y, Davenport C, Leeflang M, et al. Signs and symptoms to determine if a patient presenting in primary care or hospital outpatient settings has COVID‐19. Cochrane Database of Systematic Reviews [Internet]. 2022; Available from: <http://dx.doi.org/10.1002/14651858.CD013665.pub3>

1. Sutherland 2021

Sutherland J., Cooper-Jones B. 166 SABR FOR THE TREATMENT OF OLIGOMETASTATIC CANCER - A HEALTH TECHNOLOGY ASSESSMENT. Radiotherapy and Oncology. 2021;163:S70–1.

1. Sutton 2017

Sutton AJ. Not enough I say! Expand the remit of living systematic reviews to inform future research. Journal of clinical epidemiology. 2017;91:54–5.

1. Tan 2024

Tan J, Renton W, Whittle S, Takken T, Johnston R, Tiller G, et al. Methotrexate for juvenile idiopathic arthritis. Cochrane Database of Systematic Reviews [Internet]. 2024; Available from: http://dx.doi.org/10.1002/14651858.CD003129.pub2

1. Tercero-Hidalgo 2021

Tercero-Hidalgo JR, Khan KS, Bueno-Cavanillas A, Fernandez-Lopez R, Huete JF, Amezcua-Prieto C, et al. COVID-19 evidence syntheses with artificial intelligence: an empirical study of systematic reviews. Dryad. 2021.

1. Ters 2014

Ters P, Badgett RG. A living meta-analysis of colchicine for pericarditis. The Annals of pharmacotherapy. 2014;48:1398–9.

1. terSchure 2022

ter Schure J.A., Ly A., Belin L., Benn C.S., Bonten M.J.M., Cirillo J.D., et al. Bacillus Calmette-Guerin vaccine to reduce COVID-19 infections and hospitalisations in healthcare workers - a living systematic review and prospective ALL-IN meta-analysis of individual participant data from randomised controlled trials. medRxiv [Internet]. 2022; Available from: https://www.medrxiv.org/

1. Thakar 2023

Thakar A, Panara K, Goyal M, Kumari R, Sungchol K. AYUSH (Indian System of Medicines) Therapeutics for COVID-19: A Living Systematic Review and Meta-Analysis (First Update). JOURNAL OF INTEGRATIVE AND COMPLEMENTARY MEDICINE. 2023;29:139–55.

Thakar A, Panara K, Goyal M, Kumari R, Sungchol K. Ayush [Indian System of Medicines] Prophylaxis Against COVID-19: A Living Systematic Review and Meta-Analysis (Second Update). JOURNAL OF INTEGRATIVE AND COMPLEMENTARY MEDICINE. 2024;

1. Theodora 2020

Theodora O, Boutron I, Pierre O, Cabanac G, Ravaud P. Dataset for article: Changes in evidence for studies assessing interventions for COVID-19 reported in preprints: meta-research study. BMC Med 18, 402 (2020). Zenodo. 2020;

1. Thwaites 2017

Thwaites J. Digital guidelines: An international perspective. Asia-Pacific Journal of Clinical Oncology. 2017;13:104.

1. Tiller 2022

Tiller G, Renton WD, Tan J, Whittle S, Avery J, Munro J, et al. Modified Delphi study to identify priority clinical questions for the Australian living guidelines for the management of Juvenile Idiopathic Arthritis. Pediatric rheumatology online journal. 2022;20:52.

1. Tleyjeh 2021

Tleyjeh IM, Kashour Z, Riaz M, Hassett L, Veiga VC, Kashour T. Efficacy and safety of tocilizumab in COVID-19 patients: a living systematic review and meta-analysis-first update. Clinical microbiology and infection : the official publication of the European Society of Clinical Microbiology and Infectious Diseases [Internet]. 2021; Available from: <http://ovidsp.ovid.com/ovidweb.cgi?T=JS&PAGE=reference&D=medp&NEWS=N&AN=33915284>

1. Tonia 2023

Tonia T, Buitrago-Garcia D, Peter N, Mesa-Vieira C, Li T, Furukawa T, et al. Tool to assess risk of bias in studies estimating the prevalence of mental health disorders (RoB-PrevMH). BMJ MENTAL HEALTH. 2023;26.

1. Tort 2021

Tort S, Burch J. For people with moderate to severe COVID‐19, what are the benefits and harms of vitamin D supplementation? Cochrane Clinical Answers [Internet]. 2021; Available from: <http://dx.doi.org/10.1002/cca.3689>

1. Tortosa 2023

Tortosa F, Donato M, Torales S, Malla C, Uribe J, Izcovich A. Evidence synthesis for COVID-19 interventions: update reports from Argentina’s National Commission for Health Technology Assessment. REVISTA PANAMERICANA DE SALUD PUBLICA-PAN AMERICAN JOURNAL OF PUBLIC HEALTH. 2023;47.

1. Tunnicliffe 2020

Tunnicliffe DJ, Cashmore B, Saglimbene VM, Palmer S, Craig JC, Johnson DW, et al. Hmg coa reductase inhibitors (statins) for people with chronic kidney disease not requiring dialysis: A living cochrane review. Nephrology. 2020;25:29.

Tunnicliffe D, Palmer S, Cashmore B, Saglimbene V, Krishnasamy R, Lambert K, et al. HMG CoA reductase inhibitors (statins) for people with chronic kidney disease not requiring dialysis. Cochrane Database of Systematic Reviews [Internet]. 2023; Available from: http://dx.doi.org/10.1002/14651858.CD007784.pub3

1. Turki 2018

Turki H. Citation analysis is also useful to assess the eligibility of biomedical research works for inclusion in living systematic reviews. Journal of clinical epidemiology. 2018;97:124–5.

1. Urrea 2020

Urrea G, Llanos P, Meza N, Vargas M, Ortiz-Munoz LE, Rada G, et al. Nonsteroidal anti-inflammatory drugs in patients with COVID-19: A living systematic review. Open Science Framework. 2020;

1. Van der Vlist 2021

van der Vlist AC, Winters M, Verhaar JAN, de Vos R-J, Weir A, Ardern CL, et al. Which treatment is most effective for patients with Achilles tendinopathy? A living systematic review with network meta-analysis of 29 randomised controlled trials. British journal of sports medicine. 2021;55:249–56.

1. VanBaar 2022

Van Baar J., Kostova E., Van Wely M. COVID-19 in pregnant women: a living systematic review and meta-analysis on the risk and prevalence of pregnancy loss. Human Reproduction. 2022;37:i80.

1. Verdejo 2020

Verdejo C, Vergara-Merino L. Macrolides for the treatment of COVID-19: A living systematic review. Open Science Framework. 2020;

1. Verdugo-Paiva 2020a

Verdugo F, Acuna MP, Sola I, Rada G. Remdesivir for the treatment of COVID-19: A living systematic review. Open Science Framework. 2020;

Verdugo-Paiva F, Acuna MP, Sola I, Rada G. Remdesivir for the treatment of COVID-19: a living systematic review, Remdesivir for the treatment of COVID-19: a living systematic review. Medwave. 2020;20:e8080.

1. Verdugo-Paiva 2020b

Verdugo-Paiva F, Izcovich A, Ragusa M, Rada G. Lopinavir-ritonavir para COVID-19: una revision sistematica viva, Lopinavir-ritonavir for COVID-19: A living systematic review. Medwave. 2020;20:e7967.

1. Vieira 2023

Vieira A, Wu N, Joyal-Desmarais K, Sanuade C, Ribeiro P, Stojanovic J, et al. DOES THE EFFECTIVENESS OF BOOSTER COVID-19 VACCINES CHANGE OVER TIME? RESULTS FROM A LIVING EVIDENCE SYNTHESIS FOCUSED ON THE OMICRON PERIOD. INTERNATIONAL JOURNAL OF BEHAVIORAL MEDICINE. 2023;30:S54–5.

1. Vints 2023

Vints W, Gökçe E, Langeard A, Pavlova I, Çevik Ö, Ziaaldini M, et al. Myokines as mediators of exercise-induced cognitive changes in older adults: protocol for a comprehensive living systematic review and meta-analysis. FRONTIERS IN AGING NEUROSCIENCE. 2023;15.

1. Vogel 2020

Vogel JP, Tendal B, Giles M, Whitehead C, Burton W, Chakraborty S, et al. Clinical care of pregnant and postpartum women with COVID-19: Living recommendations from the National COVID-19 Clinical Evidence Taskforce. The Australian & New Zealand journal of obstetrics & gynaecology. 2020;60:840–51.

1. Vyas 2024

Vyas N, Bennett A, Shaver N, Beck A, Zitiktye G, Whelan B, et al. SARS-CoV-2 transmission risk for common group activities and settings: a living scoping review. EUROPEAN JOURNAL OF PUBLIC HEALTH. 2024;34:196–201.

1. Wagner 2021

Wagner C, Griesel M, Mikolajewska A, Mueller A, Nothacker M, Kley K, et al. Systemic corticosteroids for the treatment of COVID‐19. Cochrane Database of Systematic Reviews [Internet]. 2021; Available from: <http://dx.doi.org/10.1002/14651858.CD014963>

Wagner C., Griesel M., Mikolajewska A., Metzendorf M.-I., Fischer A.-L., Stegemann M., et al. Systemic corticosteroids for the treatment of COVID-19: Equity-related analyses and update on evidence. Cochrane Database of Systematic Reviews. 2022;2022:CD014963.

1. Wang 2021

Wang Q., Hou L.-Y., Zhu H.-F., Li M.-T., Zhang Q., Zhou Q., et al. Proposal of Living Evidence-based Guideline for Combination of Traditional Chinese and Western Medicine for Treatment of COVID-19. Zhongguo Zhongyao Zazhi. 2021;46:5117–22.

1. Wang 2023

Wang Q, Wang J, Wu R, Li Y, Yao C, Xie F, et al. The safety of digestive tract cancer surgery during COVID-19: A living systematic review and meta-analysis. ASIAN JOURNAL OF SURGERY. 2023;46:4138–51.

1. Webster 2021

Webster KE, O’Byrne L, MacKeith S, Philpott C, Hopkins C, Burton MJ. Interventions for the prevention of persistent post-COVID-19 olfactory dysfunction. The Cochrane database of systematic reviews. 2021;7:CD013877.

Webster KE, MacKeith S, Philpott C, Hopkins C, Burton MJ. Interventions for the prevention of persistent post-COVID-19 olfactory dysfunction. Cochrane Database of Systematic Reviews. 2021;2021:CD013877.

Webster KE, O’Byrne L, MacKeith S, Philpott C, Hopkins C, Burton MJ. Interventions for the prevention of persistent post-COVID-19 olfactory dysfunction. The Cochrane database of systematic reviews. 2022;9:CD013877.

1. Whittle 2020

Whittle S, Buchbinder R, Thomas M, Hazlewood G, Kamso MM, Pardo JP. A living network meta-analysis of treatments for rheumatoid arthritis: Novelty at the frontier of living evidence. Internal Medicine Journal. 2020;50:39.

1. Wieland 2021

Wieland L.S. Vitamin D supplementation for the treatment of COVID-19: Summary of a living Cochrane review. Explore. 2021;17:481–2.

1. Williams 2023

Williams M, Vogel J, Gallos I, Ramson J, Chou D, Oladapo O. The use of network meta-analysis in updating WHO living maternal and perinatal health recommendations. BMJ GLOBAL HEALTH. 2023;8.

1. Winters 2021

Winters M, Holden S, Lura CB, Welton NJ, Caldwell DM, Vicenzino BT, et al. Comparative effectiveness of treatments for patellofemoral pain: a living systematic review with network meta-analysis. BRITISH JOURNAL OF SPORTS MEDICINE. 2021;55:369-+.

1. Wolf 2021

Wolf V., Kuhnel A., Teckentrup V., Koenig J., Kroemer N.B. Does transcutaneous auricular vagus nerve stimulation affect vagally mediated heart rate variability? A living and interactive Bayesian meta-analysis. Psychophysiology. 2021;58:e13933.

1. Wu 2023a

Wu N, Joyal-Desmarais K, Ribeiro P, Vieira A, Stojanovic J, Sanuade C, et al. Long-term effectiveness of COVID-19 vaccines against infections, hospitalisations, and mortality in adults: findings from a rapid living systematic evidence synthesis and meta-analysis up to December, 2022. LANCET RESPIRATORY MEDICINE. 2023;11:439–52.

1. Wu 2023b

Wu N, Joyal-Desmarais K, Vieira A, Sanuade C, Jagwani M, Paquet L, et al. COVID-19 boosters versus primary series: update to a living review. LANCET RESPIRATORY MEDICINE. 2023;11:E87–8.

1. Xu 2020

Xu W, He Y, Lang Z, Li X, Dozier M, Kirolos A, et al. What is the evidence for transmission of COVID-19 by children in schools? A living systematic review. Journal of global health. 2020;10:021104.

Xu W., Li X., Dong Y., Dozier M., He Y., Kirolos A., et al. SARS-CoV-2 transmission in schools: An updated living systematic review (version 2; November 2020). Journal of global health. 2021;11:10004.

1. Ye 2022

Ye J., Sinnathurai P., Hill C., Barrett C., Hardy L., Buchbinder R., et al. ENGAGING CLINICIANS AND CONSUMERS IN THE DESIGN AND IMPLEMENTATION OF A NATIONAL EDUCATION PROGRAM FOR INFLAMMATORY ARTHRITIS. Internal Medicine Journal. 2022;52:31.

1. Yiu 2022

Yiu Z, Chi C, Ingram J, Flohr C. Checking for update ... living systematic reviews and clinical practice guidelines in the BJD. BRITISH JOURNAL OF DERMATOLOGY. 2022;186:761–2.

1. Yu 2023a

Yu S, Choi M, Cheong C, Ryoo S, Huh K, Yoon Y, et al. Clinical efficacy and safety of SARS-CoV-2-neutralizing monoclonal antibody in patients with COVID-19: A living systematic review and meta-analysis. JOURNAL OF MICROBIOLOGY IMMUNOLOGY AND INFECTION. 2023;56:909–20.

1. Yu 2023b

Yu S, Choi M, Ryoo S, Cheong C, Huh K, Yoon Y, et al. Clinical efficacy of inhaled corticosteroids in patients with coronavirus disease 2019: A living review and meta-analysis. PLOS ONE. 2023;18.

1. Zampieri 2022a

Zampieri F.G., Cavalcanti A.B., Di Tanna G.L., Damiani L.P., Hammond N.E., Machado F.R., et al. Statistical Analysis Plan for Balanced versus Saline trialists living systematic review individual patient and aggregated data meta-analysis of randomized controlled trials (BEST-Living Study). medRxiv [Internet]. 2022; Available from: https://www.medrxiv.org/

1. Zampieri 2022b

Zampieri FG, Cavalcanti AB, Di Tanna GL, Damiani LP, Hammond NE, Machado FR, et al. Protocol for balanced versus saline trialists: living systematic review and individual patient data meta-analysis of randomised controlled trials (BEST-Living study). Critical care and resuscitation : journal of the Australasian Academy of Critical Care Medicine. 2022;24:128–36.

1. Zeiler 2021

Zeiler FA, McFadyen C, Newcombe VFJ, Synnot A, Donoghue EL, Ripatti S, et al. Genetic Influences on Patient-Oriented Outcomes in Traumatic Brain Injury: A Living Systematic Review of Non-Apolipoprotein E Single-Nucleotide Polymorphisms. Journal of neurotrauma. 2021;38:1107–23.

1. Zeraatkar 2022

Zeraatkar D, Pitre T, Leung G, Cusano E, Agarwal A, Khalid F, et al. Consistency of covid-19 trial preprints with published reports and impact for decision making: retrospective review. BMJ medicine. 2022;1:e000309.

1. Zhang 2021

Zhang X., Shang L., Fan G., Gu X., Xu J., Wang Y., et al. The Efficacy and Safety of Janus Kinase Inhibitors for Patients With COVID-19: A Living Systematic Review and Meta-Analysis. Frontiers in Medicine. 2022;8:800492.

Zhang X, Shang L, Fan G, Gu X, Xu J, Wang Y, et al. Data_Sheet_1_The Efficacy and Safety of Janus Kinase Inhibitors for Patients With COVID-19: A Living Systematic Review and Meta-Analysis.pdf. Figshare. 2022.

1. Zhao 2022

Zhao J, Bai W, Zhang Q, Su Y, Wang J, Du X, et al. Evidence-based practice implementation in healthcare in China: a living scoping review. The Lancet regional health Western Pacific. 2022;20:100355.

1. Zhelnov 2020

Zhelnov P. Zheln PRISMA Living Report. Open Science Framework. 2020;

Zhelnov P. Zheln.com: A universal living overview of health-related systematic reviews. Open Science Framework. 2020;

# Articles excluded because of they provide insights that were only related to living guidelines

1. Akl 2017

Akl EA, Meerpohl JJ, Elliott J, Kahale LA, Schunemann HJ, Living Systematic Review N, et al. Living systematic reviews: 4. Living guideline recommendations. Journal of clinical epidemiology. 2017;91:47–53.

1. Aldin 2021

Aldin A., Follmann M., Borchmann P., Baues C., Sasse S., Brockelmann P.J., et al. German evidence-based living guideline on diagnosis, therapy and follow-up of adult patients with hodgkin lymphoma. HemaSphere. 2021;5:356.

1. Bartoszko 2021

Bartoszko JJ, Siemieniuk RAC, Kum E, Qasim A, Zeraatkar D, Ge L, et al. Prophylaxis against covid-19: living systematic review and network meta-analysis. BMJ (Clinical research ed). 2021;373:n949.

Bartoszko. Update to living systematic review on prophylaxis against covid-19. BMJ-BRITISH MEDICAL JOURNAL. 2023;380.

1. Dawson 2022

Dawson J., Sampson M., Ledoux A.A., Reed N., Zemek R. Major Updates in the Living Guideline for Pediatric Concussion Care to Clinical Practice Recommendations for 2021. Journal of Head Trauma Rehabilitation. 2022;37:E401–2.

1. Dignass 2021

Dignass A, Kucharzik T. S3-Guideline for Ulcerative colitis - Living Guideline: Review 2021. Zeitschrift fur Gastroenterologie. 2021;59.

1. Elmikati 2022

El Mikati I.K., Khabsa J., Harb T., Khamis M., Agarwal A., Pardo-Hernandez H., et al. A Framework for the Development of Living Practice Guidelines in Health Care. Annals of internal medicine. 2022;

1. Ge 2021

Ge L., Zhu H., Wang Q., Li M., Cai J., Chen Y., et al. Integrating Chinese and western medicine for COVID-19: A living evidence-based guideline (version 1). Journal of Evidence-Based Medicine. 2021;14:313–32.

1. Hill 2022

Hill K., English C., Campbell B.C.V., McDonald S., Pattuwage L., Bates P., et al. Feasibility of national living guideline methods: The Australian Stroke Guidelines. Journal of Clinical Epidemiology. 2022;142:184–93.

1. Lamontagne 2020

Lamontagne F, Agoritsas T, Macdonald H, Leo Y-S, Diaz J, Agarwal A, et al. A living WHO guideline on drugs for covid-19. Bmj-British Medical Journal [Internet]. 2020;370. Available from: <https://www.bmj.com/content/bmj/370/bmj.m3379.full.pdf>

1. Lamontagne 2021

Lamontagne F, Agoritsas T, Siemieniuk R, Rochwerg B, Bartoszko J, Askie L, et al. A living WHO guideline on drugs to prevent covid-19. BMJ (Clinical research ed). 2021;372:n526.

1. Malin 2022

Malin J.J., Spinner C.D., Janssens U., Welte T., Weber-Carstens S., Schalte G., et al. Key summary of German national treatment guidance for hospitalized COVID-19 patients: Key pharmacologic recommendations from a national German living guideline using an Evidence to Decision Framework (last updated 17.05.2021). Infection. 2022;50:93–106.

1. Millard 2022

Millard T., Elliott J.H., Green S., Tendal B., Vogel J.P., Norris S., et al. Awareness, value and use of the Australian living guidelines for the clinical care of people with COVID-19: an impact evaluation. Journal of Clinical Epidemiology. 2022;143:11–21.

1. NatlCOVID19ClinicalEvidenceTa 2024

Natl COVID 19 Clinical Evidence Ta, Millard T, Elliott J, Green S, McGloughlin S, Turner T. Exploring the use and impact of the Australian living guidelines for the clinical care of people with COVID-19: where to from here? JOURNAL OF CLINICAL EPIDEMIOLOGY. 2024;166.

1. Tendal 2021

Tendal B, McDonald S, Cumpston M, White H, Leder K, Murano M, et al. Weekly updates of national living evidence-based guidelines: methods for the Australian living guidelines for care of people with COVID-19. Journal of clinical epidemiology. 2021;131:11–21.

1. Vogel 2020

Vogel JP, Oladapo OT. Developing and applying a living guidelines approach to who maternal and perinatal health recommendations. Journal of paediatrics and child health. 2020;56:49–50.

1. Wiles 2024

Wiles L, Hibbert P, Zurynski Y, Smith C, Arnolda G, Ellis L, et al. Is it possible to make “living” guidelines? An evaluation of the Australian Living Stroke Guidelines. BMC HEALTH SERVICES RESEARCH. 2024;24.
