# Supplementary material for: A living critical interpretive synthesis to yield a framework on the production and dissemination of living evidence syntheses for decision-making
Source: Implement Sci. 2024 Sep 27;19:67. doi: 10.1186/s13012-024-01396-2 (PMC11429155; doi:10.1186/s13012-024-01396-2)
Supplement: Supplementary file 5 — Additional file 5. Detailed description of each thematic category. Detailing and explaining how each thematic category and sub-theme are framed. [file 13012_2024_1396_MOESM5_ESM.docx]

**Additional table 5.1. Thematic category 1: definition of a living evidence synthesis**

|  | **Sub-theme 1. Living evidence syntheses** | **Sub-theme 2. Updates** | **Sub-theme 3. Labelling ‘living’** |
| --- | --- | --- | --- |
| **Short description** | Understanding what constitutes a living synthesis | Understanding what constitutes an 'update' in the context of an LES? | What do we understand by the label ‘living’ in the context of evidence syntheses |
| **Details** | An LES understood as a summary of research that:   - summarizes all the existing evidence in a cumulative way; - at any defined point in time is up-to-date (i.e., it is continually updated as evidence becomes available); - can address single comparisons or multiple available comparisons (i.e., living network meta-analysis); - [similar to a non-living evidence synthesis] can use different type of methods to combine studies (narrative summary, meta-analysis, network meta-analysis); - can use different channels to be disseminated, but it would most likely have an online up-to-date summary available. | An update can be understood as any change in an evidence synthesis that:   - adds new evidence that was not previously included, coming from:   - new research that fits eligibility criteria that was made available since the last search date;   - new research that adds new interventions/comparisons for a given condition (i.e., in a living network meta-analysis);   - research that was available before the last search date, but it was not included in the original document because of:     - limitation in the search strategy/screening process;     - research was produced before, but was made available after the search date (e.g., historical material released at a specific time); - changes the eligibility criteria (and the protocol); - updates search strategies (i.e., living search strategy); - changes the presentation details of the synthesis (e.g., living document).   In studies addressing what should be considered an update in the context of non-living evidence syntheses, there is a contested definition of what should be considered an update (i.e., whether only adding new evidence should be considered a real update). | Different approaches to label as ‘living’ a given evidence synthesis, including:   - understanding the label ‘living’ as a transient status (i.e., one synthesis could be living only for a specific period of time, and could stop being living at some point); - understanding the label ‘living’ as a scale rather than a status (i.e., ‘livingness’ of a synthesis); - understanding the label ‘living’ as a *a priori* commitment from synthesisers (i.e., including a clear plan on how to incorporate new evidence when it becomes available and a credible commitment about when updates will be available).   Considering whether it is easier to label an evidence synthesis as non-living or developing criteria for an evidence synthesis to be out-of-date |

**Additional table 5.2. Thematic category 2: methods to produce living evidence syntheses**

|  | **Sub-theme 1. Assessment of the need of a living evidence synthesis (or to update one)** | **Sub-theme 2. Team management** | **Sub-theme 3. Production** |
| --- | --- | --- | --- |
| **Short description** | Methods to predict whether new literature might change the findings | Methods to facilitate the team management while producing a living evidence synthesis | Methods to facilitate the production of a ‘trustworthy’ living evidence synthesis |
| **Details** | Two groups of (non-exclusive) methods that assess:   - the probability that new literature might change the existing findings (e.g., Ottawa, RAND, etc.) - how susceptible the existing findings are to be changed if new evidence arises (e.g., GRADE certainty of the evidence is low or very low for a given outcome). - how the context or the issue might change the applicability of the findings or the understanding of a giving phenomena. | Strategies to facilitate the work in teams:   - Streamline pathways, workflows, and role definitions, by setting individual small tasks that facilitate a manageable workload across time. - Outlining clear boundaries where technology can facilitate specific tasks   Strategies to ensure team sizes that would make feasible the production of a living evidence synthesis:   - Consider the type of incentives that authors might get to participate (e.g., authorship). - Having topic-oriented living evidence synthesis communities that would be ‘ready’ to take on new syntheses - Crowdsourcing - Use of trainees | Methods to facilitate searching and study selection:   - Artificial intelligence (including machine learning, or natural language processing to check if new citations are not already included), data mining and neural networks to facilitate periodic searches (which could also be complemented with semi-automated approaches) - Living collections of evidence (curated libraries or repositories for specific topics) that can include pre-print servers - Alerts from some bibliographic databases - Librarian help desks to conduct and adapt search strategies (mediated search services)   Methods to facilitate data extraction and risk-of-bias assessment:   - Artificial intelligence and machine learning (which could also be complemented with semi-automated approaches) - Linked data from studies that have already been processed as part of another synthesis   Methods to facilitate data synthesis:   - Artificial intelligence and machine learning (which could also be complemented with semi-automated approaches) - Statistical approaches to manage the increase of type I error in repeated meta-analyses (e.g., trial sequential analysis, sequential meta-analysis, Shuster method, Bayesian frameworks, ALL-IN meta analyses, etc.) - Text templates for synthesizing evidence. |

**Additional table 5.3. Thematic category 3: when to produce a living evidence synthesis**

|  | **Sub-theme 1. Type of decisions** | **Sub-theme 2. Demand side triggers** | **Sub-theme 3. Supply side triggers** | **Sub-theme 4. Other elements to consider** |
| --- | --- | --- | --- | --- |
| **Short description** | Alternatives for an evidence producer when starting a new evidence synthesis | ‘Triggers’ when a living evidence synthesis might need to be produced | | Other elements to be considered when making a decision of when a living synthesis needs to be conducted |
| **Details** | At any given time, an evidence synthesis producer has the following options:   - starting an evidence synthesis (regardless of whether it will become living or not) – i.e., ‘baseline’ synthesis; - updating an existing evidence synthesis as a one-off exercise; - updating an existing evidence synthesis in a living way:   - making a non-living evidence synthesis (most commonly the ‘baseline’ synthesis) living (which is the focus of the next table);   - keeping living an existing living evidence synthesis (which is also the focus of the next table). | Demand-side triggers are determined when a topic is relevant for decision making, which could be influenced by:   - agenda-setting dynamics (coupling of problems, policies and politics streams), including the role of stakeholders that are aiming to influence decision maker agendas; - how the context and issues might influence agenda-setting dynamics (e.g., new variants emerge). - urgency of a given decision (i.e., immediate answer to pressing needs); - other factors that influence priorities in research priority setting processes (e.g., supplier induced demand, social media); - a need for having ‘living’ recommendations (or other evidence-informed products to promote the use of evidence in decision making e.g., living knowledge translation), and to provide living inputs to produce these recommendations; | Supply-side triggers could be coming from two bodies of evidence:   - existing body of evidence: susceptibility of the current findings to change if more evidence is added (i.e., certainty of the existing body of evidence, or level of saturation of a given concept, or immatureness of the existing evidence base) - New non-synthesized evidence is available and could change the current findings because:   - It will change the principal findings or summary estimates (e.g., effect estimates)   - It will change the certainty or confidence in the existing evidence (e.g., increasing precision of pooled estimates, or the coherence of the findings of multiple studies)   - It will solve a ‘lingering controversy’ for a given topic   - It might change the relevance of the existing question or issue (e.g., new political power balance, new technology that changes an acceptable control group, etc.) | Other elements might need to be considered by researchers when making decisions about starting a living evidence synthesis:   - workload and availability of the research team; - time that usually takes for a primary study to be included in an evidence synthesis; - the question has been already addressed by a different evidence synthesis; - new developing methods would allow a different analysis of the same data; - critical feedback received from evidence synthesis readers; - presence of publication bias, indicating the need to update the synthesis to reduce this potential effect (the risk of publication bias might be amplified too, since trials with positive results may be published sooner than trials with negative results). |

**Additional table 5.4. Thematic category 4: when to update a living evidence synthesis (keep a living evidence synthesis ‘living’)**

|  | **Sub-theme 1. Processed involved** | **Sub-theme 2. Triggers from the demand side** | **Sub-theme 3. Triggers from the supply side** | **Sub-theme 4. Frequency** |
| --- | --- | --- | --- | --- |
| **Short description** | Parts of an evidence synthesis that could be updated | 'Triggers’ when a living evidence synthesis might need to be updated | | Deciding how frequent a living evidence synthesis needs to be updated |
| **Details** | Depending on the demand and supply side ‘triggers’ (including the role of funders or intermediaries), for a given update to a living evidence synthesis, the following parts of the synthesis could be updated:   - eligibility criteria; - search strategies (‘adaptive’ search strategies); - data synthesis; - data appraisal; - publication. | A potential change in any of the ‘triggers’ outlined in the previous table. Common issues to consider might be:   - whether or not the research question is still a priority for decision making (e.g., whether the first version of the synthesis continues to be used frequently); - an existing synthesis has already addressed the topic. | Similar to the previous table, supply-side triggers could be coming from two bodies of evidence:   - Susceptibility of the findings from an existing body of evidence to change if more evidence is added (i.e., certainty of the existing body of evidence, or level of saturation of a given concept) - New non-synthesized evidence is frequently made available (i.e., topic remains active from a research perspective; e.g., large number of studies underway, type of policy being implemented in several places and could be evaluated), and could change the current findings because:   - It will change the summary estimates (e.g., effect estimates)   - It will change the certainty of the existing body of evidence   - It will solve a ‘lingering controversy’ in a given topic | Two main approaches could be selected to decide how frequently a living evidence synthesis will get updated:   - regular frequency (e.g., weekly, monthly, quarterly, etc.); - tailored frequency (that could also change over time), based on the occurrence of ‘triggers’ outlined in subthemes 2 and 3, and the negotiations with the demand.   A number of supportive tools can help researchers decide on defining either approach:   - methods to estimate the needed frequency are outlined in a previous table (could be done by setting a specific number or scale of new studies available to trigger a new update); - having a steering committee that can assess the ‘triggers’ and can advise researchers on what part of an evidence synthesis should be updated (sub-theme 1).   Regardless of the alternative chosen by researchers, researchers should engage in credible commitments so the frequency being communicated is the actual frequency that a living evidence synthesis is updated. |

**Additional table 5.5. Thematic category 5: dissemination of the findings of a living evidence synthesis**

|  | **Sub-theme 1. Platforms** | **Sub-theme 2. Structured format** | **Sub-theme 3. Living evidence syntheses users** | **Sub-theme 4. Speeding-up** |
| --- | --- | --- | --- | --- |
| **Short description** | Platforms that could be used to make available the findings | Adaptations to the format that can be used to streamline dissemination processes | Types of decision-makers and evidence intermediaries that can use the findings of LESs | Strategies that can be used to reduce the time from which findings are available and are used by decision-makers and evidence intermediaries |
| **Details** | Different platforms (and combinations) to make available the findings of a living evidence synthesis are available:   - website; - scientific journal; - interactive platforms (e.g., apps) and infographics.   These platforms respond differently to the multiple challenges and opportunities that a living evidence synthesis creates. Some of these challenges and opportunities are:   - Limited number of words and tables/figures to present the findings - Presenting results in alternative formats (e.g., videos) - Length of the editorial process (including peer-synthesis) - Indirect benefits associated with the reputation of publishing an article in a high-impact scientific journal - Authorship of each update - Availability of previous updates (e.g., in an appendix, different DOI, different URL, etc.) - Possibility to improve user experience with the data synthesized (e.g., interactive summary of findings tables) - Further interaction with users, so they can contribute on improving the living evidence synthesis in future updates. | A number of different parts within a living evidence synthesis could be accommodated to allow the regular updating of evidence, and allowing decision-makers and evidence intermediaries to easily find what they were looking on a given context:   - creating a section in each update that highlights the changes from the previous version; - providing clear information for users on what parts of the synthesis were updated (e.g., number of new studies added using, for example, tailored PRISMA standards, search dates, etc.), even though the findings do not change); - making explicit credible commitments about the frequency to expect a new update; - detailing a clear and explicit and transparent update plan. - Reporting why the LES is needed and needs to be updated in that frequency. | The following types of users might be using the findings of a living evidence synthesis:   - primary researchers (e.g., trialists might want to see their findings in the context of other similar studies); - intermediate users (guidelines, HTA recommendations) - decision makers.   Evidence producers might want to adapt their LES findings to improve the user experience and thereby better support their uptake  LESs can strengthen collaborations with decision-makers by explicitly involving them in co-production | A number of strategies could be used to reduce the time in which findings are available for decision-makers and evidence intermediaries (i.e., the latest version is available as quickly as possible):   - Pre-print servers to publish the non-peer synthesised results in advance; - for living evidence syntheses published in a scientific journal:   - abbreviated (or partial reports) submissions of updates (e.g., only the findings tables and abstracts were updated);   - short commentary published to flag that searches were updated;   - previous updates are kept as an appendix;   - introducing flexibility of the authorship criteria (e.g., IMJE) for allowing ‘evolving authorship’;   - identifying a pool of researchers by topic= available to act as peer synthesisers in a fast way; - notifying readers that the searches were updated, even though there were no changes to the findings. |

**Additional table 5.6. Thematic category 6: when to discontinue updates of a living evidence synthesis**

|  | **Sub-theme 1. Demand-side triggers** | **Sub-theme 2. Supply-side triggers** | **Sub-theme 3. Other elements to consider** |
| --- | --- | --- | --- |
| **Short description** | 'Triggers' when a living evidence synthesis could stop being updated | | Other elements to be considered when making a decision of when a living synthesis can stop being updated |
| **Details** | A potential change in any of the ‘triggers’ outlined in previous tables. Common issues to consider might be:   - whether or not the research question is still a priority for decision making or if it can be re-framed; - the issue might not be a priority anymore, but it could have strategic importance (e.g., pandemic preparedeness). | A potential change in any of the ‘triggers’ outlined in previous thematic categories. Common issues to consider might be:   - the findings using the latest body of evidence are unlikely to change (e.g., high certainty/conclusiveness of the evidence, reached saturation); - no new evidence is expected to be available. | Other elements that might be considered are:   - engagement of the synthesis team with the process and final product (e.g., website, journal publication, etc.); - planned obsolescence of a funded project; - contextual variables, (e.g., alternative solutions to address the same issue are available, availability of vaccines made natural immunity questions less important). |
